# Antiproliferative Triterpenoid Saponins from *Leptaulus citroides* Baill. from the Madagascar Rain Forest

**DOI:** 10.1007/s13659-015-0083-1

**Published:** 2016-01-08

**Authors:** Qingxi Su, Peggy J. Brodie, Yixi Liu, James S. Miller, Naina M. Andrianjafy, Rabodo Antsiferana, Vincent E. Rasamison, David G. I. Kingston

**Affiliations:** Department of Chemistry, Virginia Tech Center for Drug Discovery, M/C 0212, Virginia Tech, Blacksburg, VA 24061 USA; Missouri Botanical Garden, P.O. Box 299, St. Louis, MO 63166-0299 USA; Missouri Botanical Garden, B.P 3391, 101 Antananarivo, Madagascar; Centre National d’Application des Recherches Pharmaceutiques, B.P 702, 101 Antananarivo, Madagascar

**Keywords:** Antiproliferative activity, Triterpenoid saponins, *Leptaulus citroides***(**Cardiopteridacea)

## Abstract

**Abstract:**

Bioassay-guided fractionation of EtOH extracts obtained from the roots and wood of the Madagascan plant *Leptaulus citroides* Baill. (Cardiopteridaceae) led to the isolation of ethyl esters of three new triterpenoid saponins (**1**–**3**) and the known sesquiterpenoid cinnamosmolide (**4**). The structures of **1**–**3** were elucidated by extensive 1D and 2D NMR experiments and mass spectrometry. Compounds **1**, **2**, and **4** showed moderate cytotoxicity against the A2780 human ovarian cancer cell line with IC_50_ values of 2.8, 10.2 and 2.0 µM, respectively.

**Graphical Abstract:**

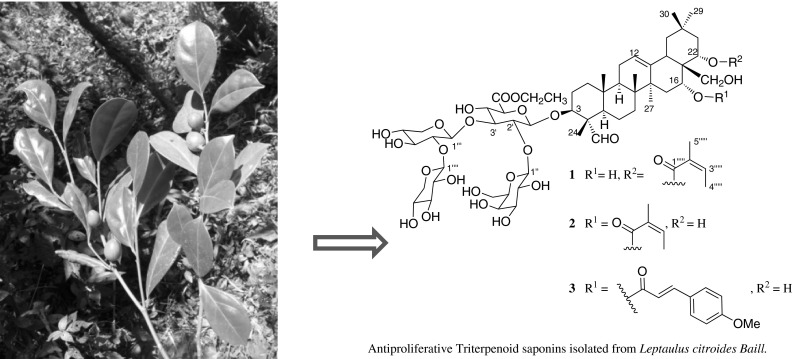

**Electronic supplementary material:**

The online version of this article (doi:10.1007/s13659-015-0083-1) contains supplementary material, which is available to authorized users.

## Introduction

In our continuing search for bioactive natural products from Madagascar rainforests as part of the International Cooperative Biodiversity Group (ICBG) project, we have focused on the isolation of antiproliferative natural products from plant extracts collected from Madagascar forests [2, 3]. As part of the research, we selected the EtOH extracts of the roots and wood of the plant *Leptaulus citroides* (Cardiopteridaceae) for investigation of antiproliferative natural products, since no previous chemical work has been reported on this genus. Bioassay-guided fractionation of the roots and wood extracts yielded ethyl esters of three new triterpenoid saponins (**1**–**3**) and the known sesquiterpenoid cinnamosmolide (**4**) [4]. Here we report the structure elucidation of compounds **1**–**3** and their antiproliferative activities.

## Results and Discussion

**Fig. 1:**
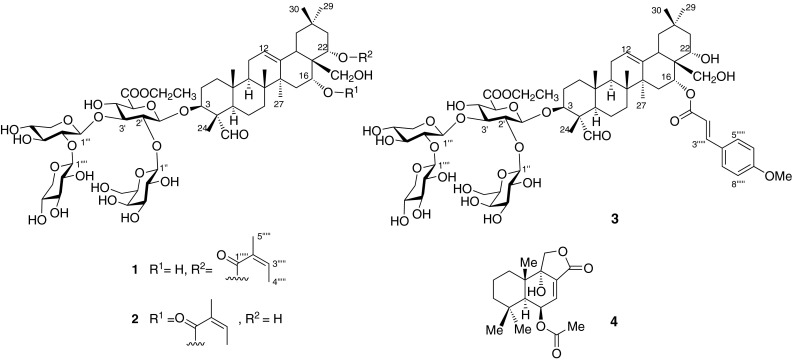
Structures of compounds **1**–**4**

Ethyl leptauloside A (**1**) was obtained as an amorphous white powder. Its HRESIMS revealed a sodiated quasi-molecular ion peak at *m/z* 1223.5806 [M + Na]^+^, corresponding to the molecular formula C_59_H_92_O_25_. Compound **1** was assigned as an olean-12-ene triterpene derivative based on its 1D and 2D NMR spectra. The ^1^H NMR spectrum of the aglycone part of compound **1** displayed characteristic signals of six singlet methyl groups [*δ*_H_ 0.91, 0.95, 1.03, 1.05, 1.16 and 1.50, (each 3H, all s, 29, 26, 25, 30, 24, 27-H_3_)], an olefin group (*δ*_H_ 5.35, t, *J* = 3.5 Hz, H-12) and an aldehyde group (*δ*_H_ 9.44, s). The position of the olefin group was confirmed by HMBC correlations of H_3_-27 (*δ*_H_ 1.50, s) to C-13 (*δ*_C_ 144.1), and H-12 to C-11 (*δ*_C_ 24.6) [5]. The aglycone moiety was oxygenated at C-3, 16, 22, and 28, based on the HMBC correlations between H_3_-24 (*δ*_H_ 1.16, s) and C-3 (*δ*_C_ 86.3); H-22/H_3_-29/H_3_-30(*δ*_H_ 5.44, dd, *J* = 12.1, 5.6 Hz/0.91 s/1.05 s) and C-21 (*δ*_C_ 42.1); between H-16/H-22 (*δ*_H_ 4.11, brs/*δ*_H_ 5.44, dd, *J* = 12.1, 5.6 Hz) and C-17 (*δ*_C_ 45.3); and between H_2_-28 (*δ*_H_ 3.05, d, *J* = 10.9 Hz/3.25 m) and C-22 (*δ*_C_ 73.8). The aldehyde group [(*δ*_H_/*δ*_C_): 9.44, s/210.6] was located at C-4 based on the HMBC correlation between the aldehyde proton and C-24 (*δ*_C_ 10.8), in addition to comparison of 1D NMR data of **1** with previously reported data of similar compounds [6]. The 1D NMR and HSQC spectra of compound **1** showed the characteristic chemical shifts and coupling patterns of an angeloyl group, with one olefinic proton [*δ*_H_ 6.07 (1H, qq, *J* = 7.3, 1.5 Hz, H-3′′′′′)], two methyl groups [*δ*_H_ 1.98 (3H, dq, *J* = 7.3, 1.5 Hz, H_3_-4′′′′′) and 1.90 (3H, m, H_3_-5′′′′′)], a carboxyl carbon (*δ*_C_ 168.8, C-1′′′′′) and two *sp*^2^ carbons (*δ*_C_ 138.1 and 130.0, C-3′′′′′ and C-2′′′′′), in agreement with the NMR data of related compounds [5–7]. The angeloyl group was assigned to C-22 based on the HMBC correlation between H-22 and the angeloyl carbonyl carbon. The spectroscopic data of the aglycone of **1** showed good agreement with the data of similar compounds reported previously [6–10]. The relative configurations of the aglycone and tetrasaccharide moiety were determined from coupling constants and ROESY correlations. The aldehyde group at C-4 was assigned the α-equatorial orientation on the basis of ROESY correlations of H_3_-24 (*δ*_H_ 1.16, s) to the *β*-axially oriented H_3_-25 (*δ*_H_ 1.03, s) and of H-23 (*δ*_H_ 9.44, s) to H-3 (*δ*_H_ 3.87, m) and H-5 (*δ*_H_ 1.35, m), both of which were *β*-axial (Fig. 1). H-16 (*δ*_H_ 4.11, brs) was assigned a *β*-equatorial orientation based on its appearance as a broad singlet, indicating small coupling constants, while H-22 (*δ*_H_ 5.44, dd, *J* = 12.3, 5.6 Hz) had the *β*-axial orientation based on its coupling constants; these assignments were confirmed by comparison with the chemical shifts and coupling constants of related protons of apodytines A-C [8]. The relative configurations of the aglycone were supported by comparison of its ^1^H and ^13^C NMR data with those of assamsaponin A[10] and camelliasaponin B1[7].

**Fig. 2:**
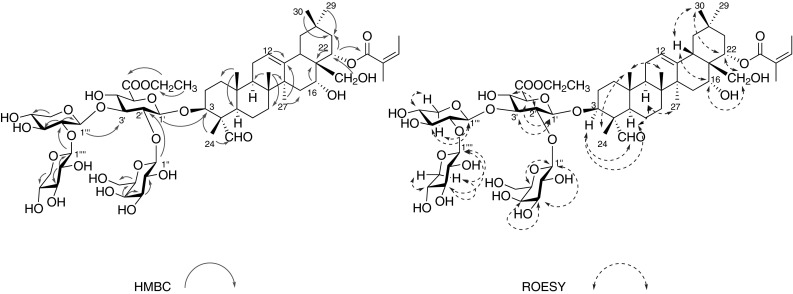
Key HMBC (*left*) and ROESY (*right*) correlations of **1**

The structure of the sugar moiety of **1** was elucidated on the bases of ^1^H-^1^H COSY, TOCSY, ROESY, HSQC, and HMBC spectra. Four anomeric protons [*δ*_H_ 4.97 (1H, d, *J* = 7.2 Hz), 4.92 (1H, d, *J* = 7.1 Hz), 4.51 (1H, d, *J* = 7.6 Hz) and 4.45 (1H, d, *J* = 7.5 Hz)] correlated with carbons at *δ*_C_ 102.7, 102.0, 107.5 and 104.8 were observed in the HSQC spectrum of **1**, indicating the presence of four sugar units. The four sugar units were identified as *β*-glucuronopyranosyl (GlcA-1′-6′), *β*-galactopyranosyl (Gal-1′′-6′′), *β*-xylopyranosyl (Xyl-1′′′-5′′′), and *β*-xylopyranosyl (Xyl-1′′′′-5′′′′), by comparison of their ^13^C NMR data with those of apodytine B [8], isotheasaponin B_1_ [11], and assamsaponin A [10]. HMBC correlations of H-1′′ (δ_H_ 4.97, d) to C-2′ (δ_C_ 77.8), H-1′′′ (δ_H_ 4.92, d) to C-3′ (*δ*_C_ 83.6), and H-1′′′′ (*δ*_H_ 4.51, d) to C-2′′′ (*δ*_C_ 85.1) indicated the connectivity of the four sugar units (Fig. 2). The HMBC correlation of H-1′ (*δ*_H_ 4.45, d, *J* = 7.5 Hz) to C-3 (*δ*_C_ 86.3), indicated that the tetrasaccharide moiety was connected to the aglycone at C-3. An ethyl ester group was present at C-5′ (*δ*_C_ 76.5) based on the COSY correlations of H_2_-7′ (*δ*_H_ 4.22, q, *J* = 7.1 Hz) to H_3_-8′ (*δ*_H_ 1.28, t, *J* = 7.1 Hz) and HMBC correlations of H_2_-7′ (*δ*_H_ 4.22, q, *J* = 7.1 Hz) and H-5′ (*δ*_H_ 3.84, d, *J* = 8.2 Hz) to a carboxyl carbon C-6′ (*δ*_C_ 170.3). These facts led to the assignment of ethyl leptauloside A (**1**) as 3-*O*-{ethyl [*β*-d-galactopyranosyl-(1 → 2)][*β*-d-xylopyranosyl-(1 → 2)-*β*-d-xylopyranosyl-(1 → 3)]-*β*-d-glucopyranosiduronate}-(3*β*,4*α*,16*α*,22*α*)-16,28-dihydroxy-22-{[(2*Z*)-2-methyl-1-oxo-2-buten-1-yl]oxy}-23-oxoolean-12-en-3-yl.

The structures and absolute configurations of the sugar moieties of **1** were confirmed by the method described by Tanaka and Kouno [12]. Compound **1** was subjected to acid hydrolysis and the resulting mixture was derivatized by treatment with l-cysteine methyl ester and *o*-tolylisothiocyanate. Comparison of the HPLC retention times of the resulting *o*-tolylthiocarbamoyl-thiazolidine derivatives of the sugar units with those of standards prepared from l-cysteine methyl ester and d and l-glucose, d and l-galactose, d and l-xylose, d and l-arabinose and d-glucuronic acid, and from d-cysteine methyl ester and d-glucuronic acid, confirmed the structures of the carbohydrate units as d-galactose, two d-xyloses, and d-glucuronic acid.

Ethyl esters of glucuronopyranosyl derivatives are unlikely to be natural products, so the ethyl ester was probably formed as the plant material was extracted with EtOH and the solvent was evaporated. It was regrettably not feasible to collect fresh plant material and extract it with a different solvent to confirm this hypothesis.

Ethyl leptauloside B (**2**) was obtained as an amorphous white powder. The quasi-molecular ion peak at *m/z* 1223.5781 [M + Na]^+^ corresponded to a molecular formula of C_59_H_92_O_25_. Further comparison of 1D NMR data of **2** suggested it is an isomer of **1** with the same sugar moiety and a similar aglycone to that of **1**. The only difference between **1** and **2** was the position of the angeloyl and hydroxyl groups. The angeloyl group was assigned to C-16 in **2**, as indicated by the deshielded signal of H-16 (*δ*_H_ 5.63, brs), and the hydroxyl group was assigned to C-22 due to the more shielded signal of H-22 (*δ*_H_ 4.06, dd, *J* = 12.3, 5.6 Hz) compared to that of compound **1** [8, 11]. The aglycone of **2** was therefore elucidated as identical to that of theasaponin G_2_ [13]. Compound **2** contained the same sugar moieties as **1** as shown by the essentially identical 1D NMR data of the two compounds. The structure of **2** was further confirmed by comparison of HSQC, HMBC and ROESY data with those of **1**. Thus, the structure of ethyl leptauloside B (**2**) was determined as 3-*O*-{ethyl [*β*-d-galactopyranosyl-(1 → 2)][-*β*-d-xylopyranosyl-(1 → 2)-*β*-d-xylopyranosyl-(1 → 3)]-*β*-d-glucopyranosiduronate}-(3*β*,4*α*,16*α*,22*α*)-22,28-dihydroxy-16-{[(2*Z*)-2-methyl-1-oxo-2-buten-1-yl]oxy}-23-oxoolean-12-en-3-yl (Fig. 1).

Ethyl leptauloside C (**3**) was obtained as an amorphous white powder. The quasi-molecular ion peak at *m/z* 1301.5982 [M + Na]^+^ corresponded to a molecular formula of C_64_H_94_O_26_. Comparison of ^1^H and ^13^C NMR data with those of **2** indicated that they were closely related except that signals of the angeloyl group were replaced by those of a *p*-methoxycinnamoyl group in the spectra of **3**. Thus the ^1^H NMR spectrum of **3** showed signals of a *para*-substituted benzene ring at *δ*_H_ 7.54 (2H, d, *J* = 8.8 Hz) and 6.98 (2H, d, *J* = 8.8 Hz), two olefinic protons with *E*- configuration *δ*_H_ 7.75 (1H, d, *J* = 16.0 Hz) and *δ*_H_ 6.35 (1H, d, *J* = 16.0 Hz) and a methoxy group *δ*_H_ 3.84 (3H, s). These signals suggested the occurrence of a 4-methoxycinnamoyl group, and this was confirmed by HMBC correlations between the methoxy group (*δ*_H_ 3.84, s, 3H) with C7, and H_-acyl-3_ (*δ*_H_ 7.75, d, *J* = 16.0 Hz) with C_-acyl-4_ (*δ*_C_ 128.2). Comparisons of chemical shifts and coupling constants of H-16 and H-22 in **2** and **3** suggested the 4-methoxycinnamoyl group was attached to C-16 [8, 11]. Thus, the structure of ethyl leptauloside C (**3**) was determined as 3-*O*-{ethyl [*β*-d-galactopyranosyl-(1 → 2)][-*β*-d-xylopyranosyl-(1 → 2)-*β*-d-xylopyranosyl-(1 → 3)]-*β*-d-glucopyranosiduronate}-(3*β*,4*α*,6*α*, 22α)-22,28-dihydroxy-16-[(*E*-4-methoxycinnamoyl)oxy]-23-oxoolean-12-en-3-yl (Fig. 1).

Compound **4** was isolated as a white solid. Its structure was assigned as shown based on comparison of its spectroscopic data with those reported in the literature [4]. Cinnamosmolide (**4**) has also been reported to display antifungal and *α*-glucosidase inhibitory activities. [14, 15].

Compounds **1**, **2**, **3** and **4** were evaluated for antiproliferative activity against the A2780 human ovarian cancer cell line. Compounds **1**, **2** and **4** showed IC_50_ values of 2.8, 10.2, and 2.0 µM respectively, while compound **3** was inactive (IC_50_ > 20 µg/mL, inhibiting 14 % of cells growth at a concentration of 20 µg/mL) in this assay. Previous studies suggested that acylation with angeloyl groups at C-21 and C-22 can affect the biological activities of oleanane triterpenoid saponins [5, 7, 16, 17]. These results support the importance of an angeloylated 22-hydroxyl group for antiproliferative activity. The reduced activity of compound **3** compared to **2** is possibly due to the bulkiness of the 4-coumaroyl group that acylates the C-16 hydroxyl group.

## Conclusions

Ethyl esters of the three new triterpenoid saponins leptaulosides A, B and C (**1**–**3**) and the known sesquiterpenoid cinnamosmolide (**4**) were isolated from *Leptaulus citroides*. Compounds **1**, **2** and **4** showed moderate antiproliferative activity against the A2780 human ovarian cancer cell line in the A2780 assay. Ethyl leptauloside C (**3**) contained an aglycone moiety with an uncommon C-16 4-methoxycinnamate group; camelliagenin A cinnamate is one of the few examples of C-16 cinnamates of oleanane triterpenes [18].

## Experimental Section

### General Experimental Procedures

IR and UV spectra were measured on MIDAC M-series FTIR and Shimadzu UV-1201 spectrophotometers, respectively. 1D and 2D NMR spectra were recorded on a Bruker Avance 500 spectrometer in CD_3_OD; chemical shifts are given in *δ* (ppm), and coupling constants are reported in Hz. Mass spectra were obtained on an Agilent 6220 LC-TOF–MS in the positive ion mode. Optical rotations were recorded on a JASCO P-2000 polarimeter. Open column chromatography was performed using Sephadex LH-20 and silica gel (40–63 µm, Silicycle Co. USA). HPLC was performed on a Shimadzu LC-10AT instrument with a semipreparative C18 (Phenomenex Luna column, 5 µm, 250 Χ10 mm), a Shimadzu SPD M10A diode array detector, and a SCL-10A system controller. All isolated compounds were purified to 95 % purity or better, as judged by HPLC (both UV and ELSD detection) before determining bioactivity.

### Antiproliferative Bioassays

Antiproliferative activities were determined at Virginia Tech against the drug-sensitive A2780 human ovarian cancer cell line as previously described [19, 20].

### Plant Material

*Leptaulus citroides* Baill. (Cardiopteridaceae) (vernacular name Tabonaka) were collected by N. M. Andrianjafy and coworkers at an elevation of about 600 m from a 10 m tall tree. Collection was made on a slope near the town of Ambodimangavalo in the district of Vavatenina, on the d’Ihofika river near the Andranofantsona camp, at coordinates 17°39′07″S 048°58′14″E (−17.6519400, 48.9705500). Duplicate voucher specimens (Andrianjafy 323) were deposited at the Centre National d’Application des Recherches Pharmaceutiques (CNARP), the Herbarium of the Department of Forestry and Fishery Research (TEF), and the Missouri Botanical Garden, St. Louis, Missouri (MO).

### Extraction and Isolation

A ground sample of *L. citroides* bark (420 g) was extracted with EtOH (1000 mL) at room temperature to yield 23.8 g of crude EtOH extract designated MG 1619, and a 5 g portion of this extract was made available to Virginia Tech for bioassay-guided isolation. Similar treatment of the wood (412 g) (1900 mL) yielded 9.8 g of extract designated MG 1620, and a 1.7 g portion of this extract was made available to Virginia Tech for bioassay-guided isolation.

### Isolation of Bioactive Constituents

The EtOH extract of the root of *L. citroides* (MG 1619, 3 g, IC_50_ = 20 µg/mL) was suspended in aqueous MeOH (MeOH–H_2_O, 9:1, 100 mL) and extracted with hexane (5 × 100 mL portions). The aqueous fraction was then diluted to 60 % MeOH and further extracted with CH_2_Cl_2_ (5 × 100 mL portions) to give a CH_2_Cl_2_ fraction (477 mg) with an IC_50_ value of 11 µg/mL. This fraction was further subjected to size exclusion open column chromatography on Sephadex LH-20 (I.D. × L 3 × 50 cm) eluted with 1:1 CH_2_Cl_2_:MeOH to yield four fractions, of which the most active fraction F3 (168 mg) exhibited an IC_50_ of 7.9 µg/mL. Fraction F3 was applied to a silica gel column (I.D. × L 3 × 50 cm, 40–63 μm) and eluted with CHCl_3_:MeOH:H_2_O, 15:6:1 to give five fractions based on TLC profile. Fractions F3-3 (22.4 mg, IC_50_ = 2.5 µg/mL) and F3-4 (32.0 mg, IC_50_ = 9.9 µg/mL) were combined and further separated by HPLC on a semipreparative C18 column (Phenomenex Luna column, 5 µm, 25 × 1 cm) with elution by a solvent gradient from CH_3_OH:H_2_O, 50:50 to 60:40 from 0 to 10 min, to 70:30 from 20 to 30 min, to 100:0 from 30 to 35 min, ending with 100 % CH_3_OH from 35 to 45 min. This process gave crude compounds **1** (3.4 mg, t_R_ 22 min) and **2** (3.0 mg, t_R_ 23 min), and compound **3** (3.0 mg, t_R_ 26 min). Compounds **1** and **2** were each purified by HPLC on a semipreparative C18 column (Phenomenex Luna column, 5 µm, 25 × 1 cm) eluted with a same solvent gradient from CH_3_CN:H_2_O, 30:70 to 40:60 from 0 to 10 min, to 50:50 from 10 to 40 min, ending with 100 % CH_3_CN from 40 to 45 min to give purified compounds **1** (3.0 mg, t_R_ 27 min) and **2** (2.8 mg, t_R_ 40 min).

The EtOH extract of the wood of *L*. *citroides* (MG 1620, 1.5 g, IC_50_ = 20 µg/mL) was subjected to liquid–liquid partition using same procedures described above. The active dichloromethane fraction (97 mg, IC_50_ = 3.5 µg/mL) was subjected to Sephadex LH-20 (I.D. × L 3 × 50 cm) chromatography to give four fractions. The active fraction F-4b (57.5 mg, IC_50_ = 2.1 µg/mL) was then subjected to open silica gel column (I.D. × L 3 × 50 cm, 40−63 μm) eluted with CHCl_3_-MeOH, 20:1 to give four fractions. Fraction F-4b-2 (29.2 mg, IC_50_ = 1.4 µg/mL) was further separated by HPLC on a semipreparative C18 column (Phenomenex Luna column, 5 µm, 25 × 1 cm) eluted by a solvent gradient from CH_3_CN:H_2_O, 70:30 to 90:10 from 0 to 30 min, to 100:0 from 30 to 40 min, ending with 100 % CH_3_CN from 40 to 45 min. This process gave compound **4** (7.6 mg, t_R_ 32 min).

### Ethyl leptauloside A (**1**) 3-*O*-{ethyl [*β*-d-galactopyranosyl-(1 → 2)][-*β*-d-xylopyranosyl-(1 → 2)-*β*-d-xylopyranosyl-(1 → 3)]-*β*-d-glucopyranosiduronate}-(3*β*,4*α*,16*α*,22*α*)-16,28-dihydroxy-22-{[(2*Z*)-2-methyl-1-oxo-2-buten-1-yl]oxy}-23-oxoolean-12-en-3-yl

White powder; [α]_D_^23^ +3.0 (*c* = 0.19, MeOH); UV (MeOH); *λ*_max_ (log ε) 207 (6.11) nm; IR (film) ν_max_ 3350, 1773, 1613, 1521, 1158, 1078 cm^−1^. ^1^H- and ^13^C-NMR: see Table 1. HRESIMS *m/z* 1223.5806 [M + Na]^+^; C_59_H_92_O_25_Na^+^, (calc. 1223.5820).

**Table 1:** ^**1**^ **H** and ^**13**^ **C** NMR data of **1**, **2**, and **3** (CD_3_OD)

Position	**1**		**2**		**3**		Position	**1**		**2**		**3**	
	^**1**^ **H**	^**13**^ **C**	^**1**^ **H**	^**13**^ **C**	^**1**^ **H**	^**13**^ **C**		^**1**^ **H**	^**13**^ **C**	^**1**^ **H**	^**13**^ **C**	^**1**^ **H**	^**13**^ **C**
1	1.12 m, 1.73 m	39.3 CH_2_	1.12 m, 1.71 m	39.3 CH_2_	1.12 m, 1.71 m	39.3 CH_2_	2′- β- Gal						
2	1.92 m 1.77 m	25.7 CH_2_	1.95 m, 1.77 m	25.7 CH_2_	1.95 m, 1.77 m	25.7 CH_2_	1′′	4.97 d (*7.2*)	102.7 CH	4.97 d (*7.2*)	102.8 CH	4.97 d (*7.2*)	102.8 CH
3	3.87 m	86.3 CH	3.86 m	86.2 CH	3,86 m	86.2 CH	2′′	3.47 m	73.4 CH	3.46 m	73.4 CH	3.46 m	73.4 CH
4	/	56.2 C	/	56.2 C	/	56.2 C	3′′	3.49 m	75.1 CH	3.49 m	75.1 CH	3.49 m	75.1 CH
5	1.35 m	48.8 CH	1.33 m	49.0 CH	1.33 m	48.9 CH	4′′	3.80 m	71.0 CH	3.80 m	71.0 CH	3.80 m	71.0 CH
6	0.95 m, 1.54 m	21.2 CH_2_	0.95 m, 1.54 m	21.1 CH_2_	0.95 m, 1.54 m	21.1 CH_2_	5′′	3.62 m	76.6 CH	3.62 m	76.6 CH	3.62 m	76.6 CH
7	1.26 m, 1.63 m	32.5 CH_2_	1.25 m, 1.54 m	33.1 CH_2_	1.28 m, 1.56 m	33.1 CH_2_	6′′	3.71 m 3.78 m	62.5 CH_2_	3.71 m 3.78 m	62.5 CH_2_	3.71 m 3.78 m	62.5 CH_2_
8	/	42.6 C	/	41.4 C	/	41.4 C	3′- β- Xyl						
9	1.79 m	48.0 CH	1.76 m	48.0 CH	1.79 m	47.9 CH	1′′′	4.92 d (*7.1*)	102.0 CH	4.92 d (*7.1*)	102.0 CH	4.92 d (*7.1*)	102.0 CH
10	/	37.0 C	/	37.0 C	/	37.0 C	2′′′	3.39 m	85.1 CH	3.39 m	85.1 CH	3.39 m	85.1 CH
11	1.96 m, 1.80 m	24.6 CH_2_	1.95 m, 1.76 m	24.5 CH_2_	1.93 m, 1.78 m	25.4 CH_2_	3′′′	3.58 m	77.5 CH	3.58 m	77.5 CH	3.58 m	77.5 CH
12	5.35 t (*3.5)*	124.2 CH	5.37 t (*3.7*)	124.9 CH	5.39 t (*3.6)*	125.0 CH	4′′′	3.56 m	71.0 CH	3.57 m	71.0 CH	3.57 m	70.9 CH
13	/	144.1 C	/	142.7 C	/	142.8 C	5′′′	3.23 m (ax) 3.90 dd (*11.4, 5.4*) (eq)	66.7 CH_2_	3.23 m (ax) 3.90 dd (*11.4, 5.4*) (eq)	66.7 CH_2_	3.23 m (ax) 3.90 dd (*11.4, 5.4*) (eq)	66.7 CH_2_
14	/	41.8 C	/	42.5 C	/	42.6 C	2′′′- β- Xyl						
15	1.31 m, 1.73 m	35.2 CH_2_	1.42 m, 1.99 m	32.3 CH_2_	1.50 m, 1.99 m	32.3 CH_2_	1′′′′	4.51 d (*7.6*)	107.5 CH	4.51 d (*7.6*)	107.5 CH	4.50 d (*7.6*)	107.5 CH
16	4.11 brs	70.9 CH	5.63 brs	71.7 CH	5.64 brs	72.4 CH	2′′′′	3.27 m	76.2 CH	3.27 m	76.2 CH	3.27 m	76.2 CH
17	/	45.3 C	/	44.7 C	/	44.9 C	3′′′′	3.31 m	77.8 CH	3.32 m	77.8 CH	3.32 m	77.9 CH
18	2.52 brd (*12.0*)	41.3 CH	2.22 brd (*12.0*)	42.6 CH	2.26 brd (*12.0*)	42.6 CH	4′′′′	3.51 m	71.0 CH	3.51 m	71.0 CH	3.51 m	71.0 CH
19	1.06 m, 2.49 m	47.9 CH_2_	1.12 m, 2.24 dd	48.0 CH_2_	1.21 m 2.40 t (*13.5*)	48.1 CH_2_	5′′′′	3.20 m (ax) 3.96 dd (*11.4, 5.4*) (eq)	67.3 CH_2_	3.20 m 3.96 dd (*11.4, 5.4*) (eq)	67.3 CH_2_	3.20 m 3.96 dd (*11.4, 5.4*) (eq)	67.3 CH
20	/	33.2 C	/	32.1 C	/	31.8 C	Acyl						
21	1.56 m, 2.27 m	42.1 CH_2_	1.39 m, 1.67 m	44.7 CH_2_	1.46 m 1.76 m	44.6 CH_2_	1′′′′′	/	168.8 C	/	168.8 C	/	167.9 C
22	5.44 dd (*12.1, 5.6*)	73.8 CH	4.06 dd (*12.1, 5.6*)	73.8 CH	4.08 dd (*12.1, 5.6*)	73.8 CH	2′′′′′	/	130.0 C	/	129.3 C	6.35 d (*16.0*)	117.2 CH
23	9.44 s	210.6 CH	9.43 s	210.5 CH	9.41 s	210.4 CH	3′′′′′	6.07 qq (*7.3, 1.5*)	138.1 CH	6.08 qq (*7.3, 1.5*)	139.0 CH	7.75 d (*16.0*)	145.8 CH
24	1.16 s	10.8 CH_3_	1.16 s	10.8 CH_3_	1.15 s	10.8 CH_3_	4′′′′′	1.98 dq (*7.3, 1.5*)	15.9 CH_3_	1.96 dq (*7.3, 1.5*)	15.9 CH_2_	/	128.2 C
25	1.03 s	16.4 CH_3_	1.03 s	16.4 CH_3_	1.03 s	16.4 CH_3_	5′′′′′	1.90 m	20.9 CH_3_	1.99 m	21.3 CH_3_	7.54 d (*8.8*)	130.8 CH
26	0.95 s	17.3 CH_3_	0.98 s	17.2 CH_3_	0.99 s	17.2 CH_3_	6′′′′′					6.98 d (*8.8*)	115.6 CH
27	1.50 s	27.7 CH_3_	1.36 s	27.5 CH_3_	1.38 s	27.7 CH_3_	7′′′′′					/	163.3 C
28	3.05 d (*10.9*) 3.25 m	64.8 CH_2_	3.30 m, 3.62 m	70.1 CH_2_	3.23 m, 3.64 m	69.9 CH_2_	8′′′′′					6.98 d (*8.8*)	115.6 CH
29	0.91 s	33.6 CH_3_	0.94 s	33.4 CH_3_	1.02 s	34.0 CH_3_	9′′′′′					7.54 d (*8.8*)	130.8 CH
30	1.05 s	25.2 CH_3_	0.99 s	25.3 CH_3_	1.04 s	25.5 CH_3_	−OMe					3.84 s	55.9 CH_3_
3-β-GlcA													
1′	4.45 d (*7.5*)	104.8 CH	4.45 d (*7.5*)	104.8 CH	4.45 d (*7.5*)	104.8 CH							
2′	3.76 m	77.8 CH	3.76 m	77.9 CH	3.76 m	77.8 CH							
3′	3.74 m	83.6 CH	3.74 m	83.6 CH	3.74 m	83.6 CH							
4′	3.58 m	70.9 CH	3.58 m	70.9 CH	3.58 m	70.9 CH							
5′	3.84 d (*8.2)*	76.5 CH	3.84 d (*8.2)*	76.5 CH	3.84 d (*8.2)*	76.5 CH							
6′	/	170.3 C	/	170.3 C	/	170.3 C							
7′	4.22 q (*7.1*)	62.5 CH_2_	4.22 q (*7.1*)	62.5 CH_2_	4.22 q (*7.1*)	62.5 CH_2_							
8′	1.28 t (*7.1*)	14.4 CH_3_	1.28 t (*7.1*)	14.4 CH_3_	1.28 t (*7.1*)	14.4 CH_3_							

^a^Assignments based on analysis of 2D NMR spectra

^b^Data (*δ*) measured at 500 MHz and 125 MHz; *s* singlet, *br s* broad singlet, *d* doublet, *dd* doublet of doublets, *ddd* doublet of doublets of doublets, *dt* doublet of triplets, *m* multiplet. *J* values are in Hz and are omitted if the signals overlapped as multiplets. The overlapped signals were assigned from HSQC and HMBC spectra without designating multiplicity

^c^Data (*δ*) measured at 125 MHz; CH_3_, CH_2_, CH, and C multiplicities were determined by HSQC experiments

*δ* in ppm, *J* in Hz

###  Ethyl leptauloside B (**2**) 3-*O*-{ethyl [*β*-d-galactopyranosyl-(1 → 2)][-*β*-d-xylopyranosyl-(1 → 2)-*β*-d-xylopyranosyl-(1 → 3)]-*β*-d-glucopyranosiduronate}-(3*β*,4*α*,16*α*,22α)-22,28-dihydroxy-16-{[(2*Z*)-2-methyl-1-oxo-2-buten-1-yl]oxy}-23-oxoolean-12-en-3-yl

White powder; [*α*]_*D*_^23^ -7.7 (*c* = 0.18, MeOH); UV (MeOH) *λ*_max_ (log *ε*) 209 (6.11) nm; IR (film) *ν*_max_ 3378, 1773, 1613, 1521, 1078 cm^−1^. ^1^H- and ^13^C-NMR: see Table 1. HRESIMS *m/z* 1223.5781 [M + Na]^+^, C_59_H_92_O_25_Na^+^; (calc. 1223.5820).

###  Ethyl leptauloside C (**3**) 3-*O*-{ethyl [*β*-d-galactopyranosyl-(1 → 2)][-*β*-d-xylopyranosyl-(1 → 2)-*β*-d-xylopyranosyl-(1 → 3)]-*β*-d-glucopyranosiduronate}-(3*β*,4*α*,16*α*,22*α*)-22,28-dihydroxy-16-[(*E*-4-methoxycinnamoyl)oxy]-23-oxoolean-12-en-3-yl.

White Powder; [α]_D_^23^ +9.6 (*c* = 0.12, MeOH); UV (MeOH) *λ*_max_ (log ε) 330 (3.79), 282 (3.98), 225 (4.05), 209 (4.09) nm; IR (film) ν_max_ 3386, 1773, 1609, 1518, 1175, 1078, 706 cm^−1^. ^1^H- and ^13^C-NMR: see Table 1. HRESIMS *m/z* 1301.5982 [M + Na]^+^, C_64_H_94_O_26_Na^+^; (calc. 1301.5926).

###  Hydrolysis of Ethyl Leptauloside A (**1**) and Absolute Configurations of Its Carbohydrate Moieties.

Authentic methyl 2-(polyhydroxyalkyl)-3-(*o*-tolylthiocarbamoyl)-thiazolidine-4(*R*)-carboxylates were prepared from d- and l-galactose, d- and l-glucose, d- and l-xylose, d- and l-galactose, d- and l-galactose, and d-glucuronic acid by reaction with l-cysteine methyl ester and *o*-tolylisothiocyanate as described [12]. Since l-glucuronic acid was not available, the enantiomeric (o-tolylthiocarbamoyl)-thiazolidine-4(*S*)-carboxylate of d-glucuronic acid was prepared by reaction with d-cysteine methyl ester and *o*-tolylisothiocyanate. Compound **1** (2.0 mg) was treated with 3 M HCl for 4 h at 100 °C, and the solution was then neutralized with sodium bicarbonate and extracted thrice with EtOAc. The aqueous fraction was evaporated to dryness under reduced pressure. The resulting mixture of carbohydrates (0.7 mg) was then dissolved in 0.5 mL pyridine, 0.9 mg of l-cysteine methyl ester was added, and the mixture was heated at 60 °C for 1 h. *o*-Tolylisothiocyanate (0.9 mg) was then added to the mixture, which was again heated at 60 °C for 1 h. The reaction mixture was directly analyzed by reverse-phase HPLC on a Phenomenex Luna column (5 µm, 25 × 1 cm), eluted with isocratic 0.1 % formic acid in CH_3_CN/H_2_O (15:85) at a flow rate of 2.5 mL/min for 5 min, followed by 0.1 % formic acid in CH_3_CN/H_2_O (25:75) for 30 min, and a wash with 100 % CH_3_CN for 10 min. The resulting chromatogram contained three major peaks with retention times of 25.29, 29.12, and 31.25 min, identical to those of the derivatives of d-xylose, d-galactose and d-glucuronic acid. Co-injection of each of the derivatives obtained by hydrolysis of **1** with its corresponding synthetic counterpart confirmed the identity of the compounds. The peak corresponding to the d-xylose derivative was approximately twice as large as that for the d-galactose derivative, consistent with the presence of two d-xylose units in **1**.

## Electronic supplementary material

Supplementary material 1 (PDF 2013 kb)
