# Supplementary material for: Antiproliferative Triterpenoid Saponins from Leptaulus citroides Baill. from the Madagascar Rain Forest
Source: Nat Prod Bioprospect. 2016 Jan 8;6(1):31–9. doi: 10.1007/s13659-015-0083-1 (PMC4749521; doi:10.1007/s13659-015-0083-1)

SUPPLEMENTARY DATA

Antiproliferative triterpenoid saponins from *Leptaulus citroides* Baill. from the Madagascar rain forest

Qingxi Su[a], Peggy J. Brodie[a], Yixi Liu[a], James S. Miller[b], Naina M. Andrianjafy[b], Rabodo Antsiferana[c], Vincent E. Rasamison[c], and David G. I. Kingston[a,1]

[a]*Department of Chemistry and Virginia Tech Center for Drug Discovery, M/C 0212, Virginia Tech, Blacksburg, VA 24061, United States*

[b]*Missouri Botanical Garden, B.P 3391, Antananarivo 101, Madagascar*

[c]*Centre National d'Application des Recherches Pharmaceutiques, B.P 702, Antananarivo 101, Madagascar*

[*]To whom correspondence should be addressed. Tel: 540-231-6570; Fax: 540-231-3255; E-mail: dkingston@vt.edu

[1] Corresponding author. Tel.: +1 540 231 6570; fax: +1 540 231 3255.
E-mail address: dkingston@vt.edu (D.G.I. Kingston).

Experimental Section

Table 1. Retention times of the thiocarbamoyl-thiazolidine derivatives

Aldose	Absolute configuration	t_R (min)
Glactose	D	25.35
	L	26.08
Glucose	D	28.04
	L	26.76
Glucuronic acid	D	29.03
	L [a)]	28.26
Xylose	D	31.32
	L	30.02
Arabinose	D	31.71
	L	30.28

a) The t_R was obtained by using D-cysteine methyl ester

1. NMR spectra of 1

1.1 ^{1}H NMR spectrum (measured in CD_3OD)

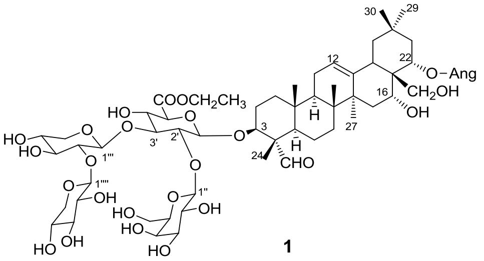

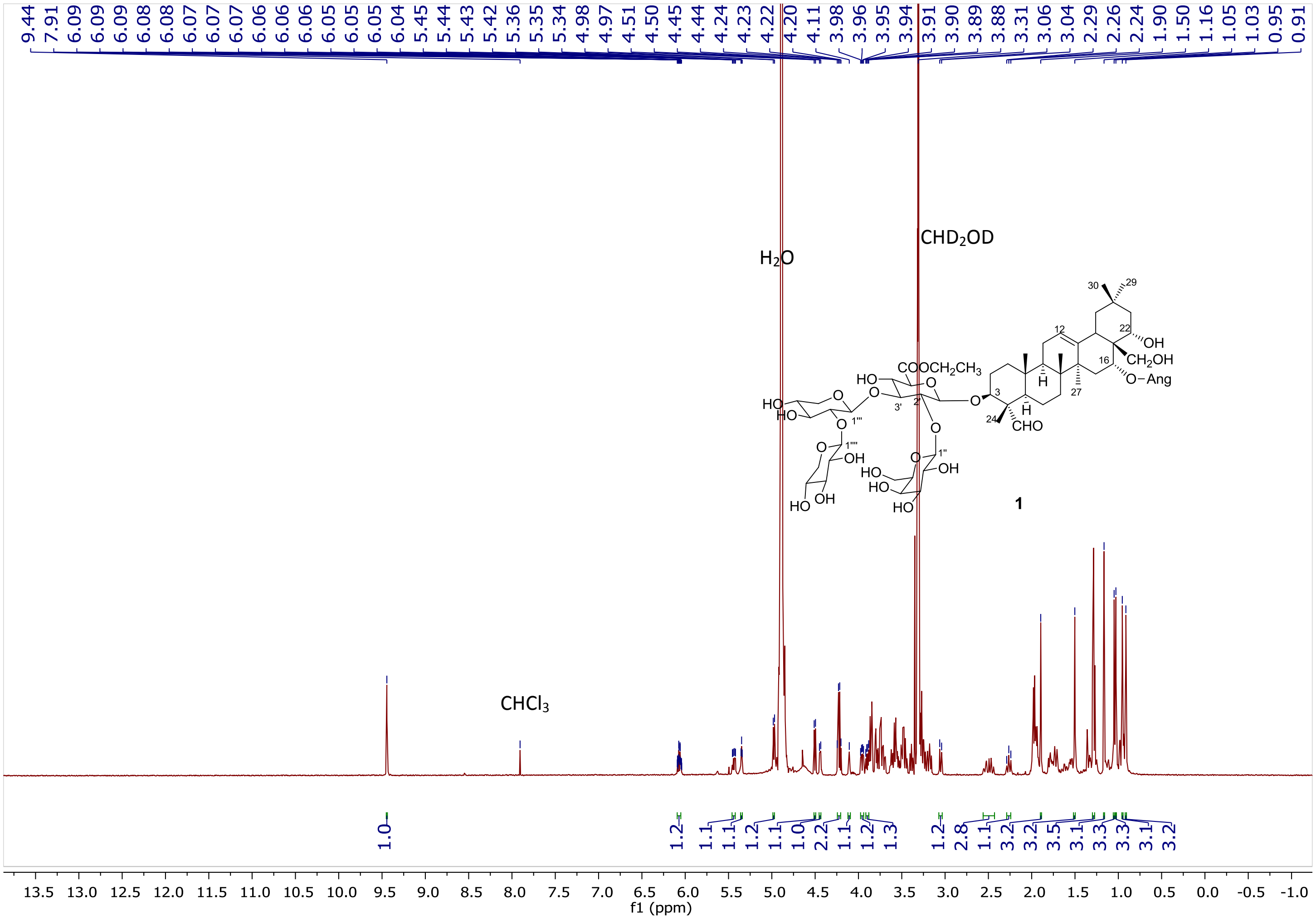

1.2 ^{13}C NMR spectrum of **1** (measured in CD_3OD)

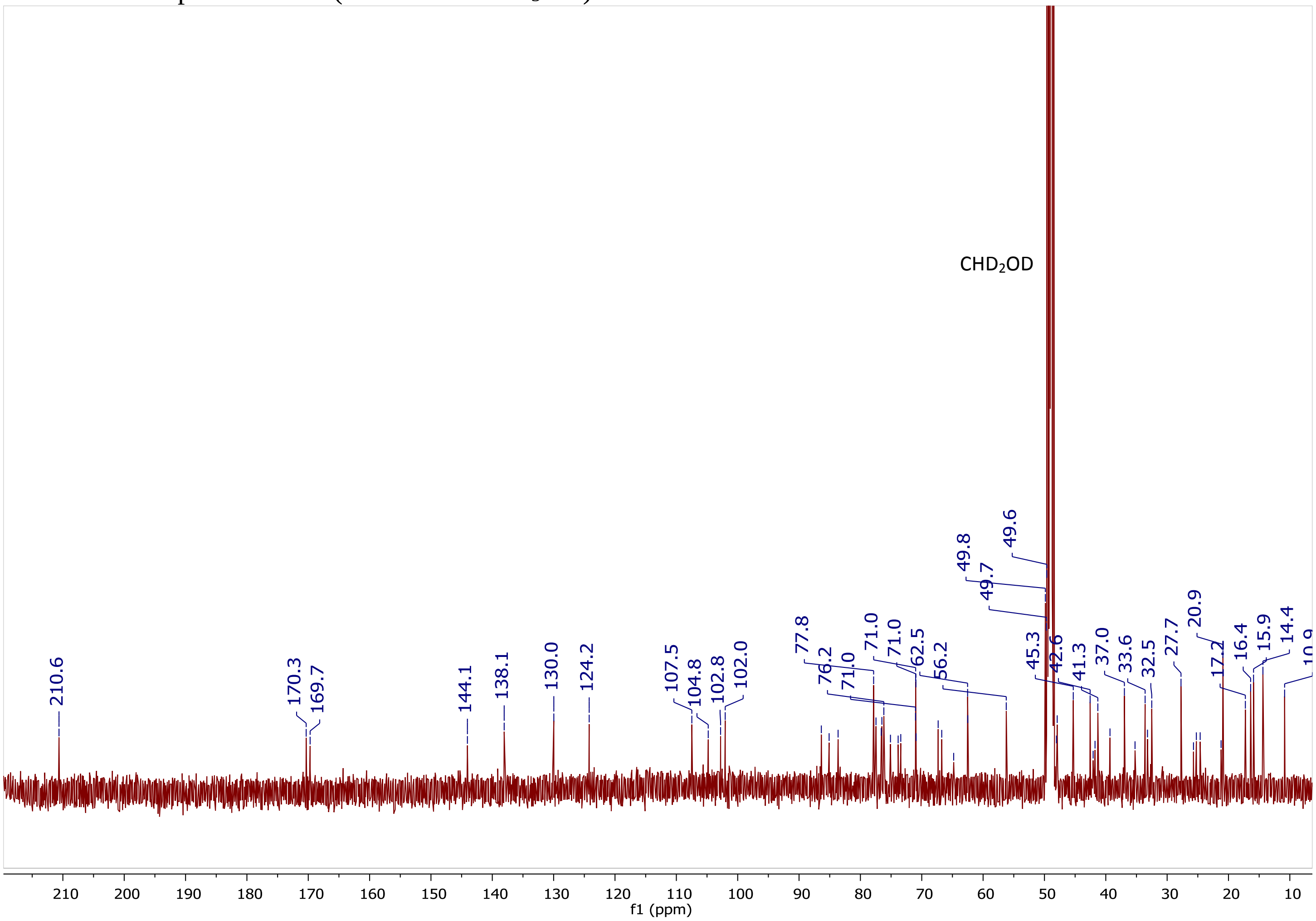

2. NMR spectra of 2

2.1 ^{1}H NMR spectrum of **2** (measured in CD_3OD)

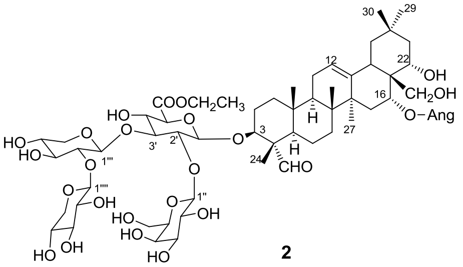

2

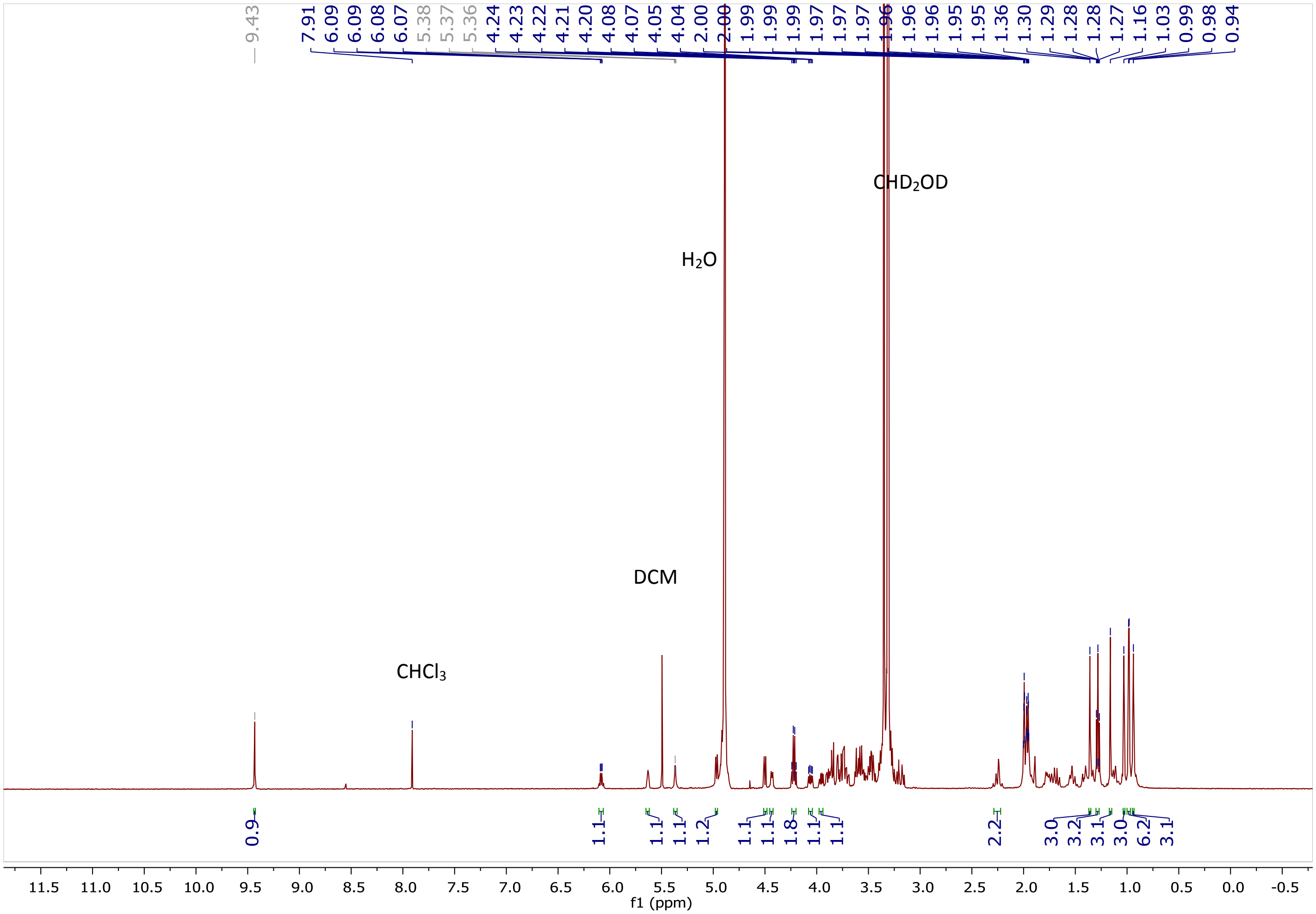

2.2 ^{13}C NMR spectrum of **2** (measured in CD_3OD)

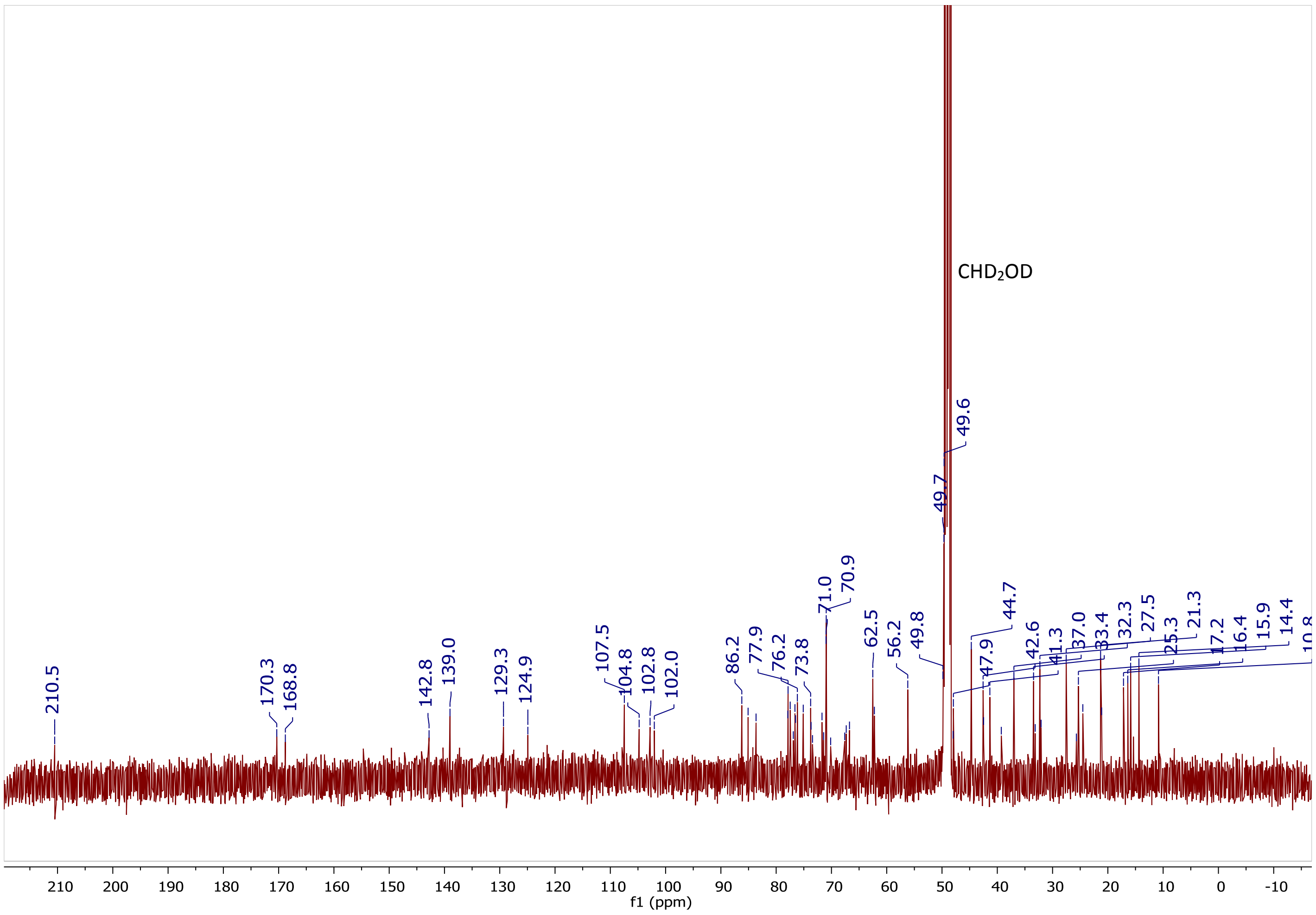

3. NMR spectra of 3

3.1 ^{1}H NMR spectrum of **3** (measured in CD_3OD)

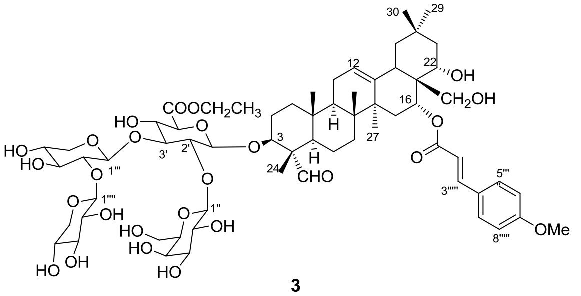

3

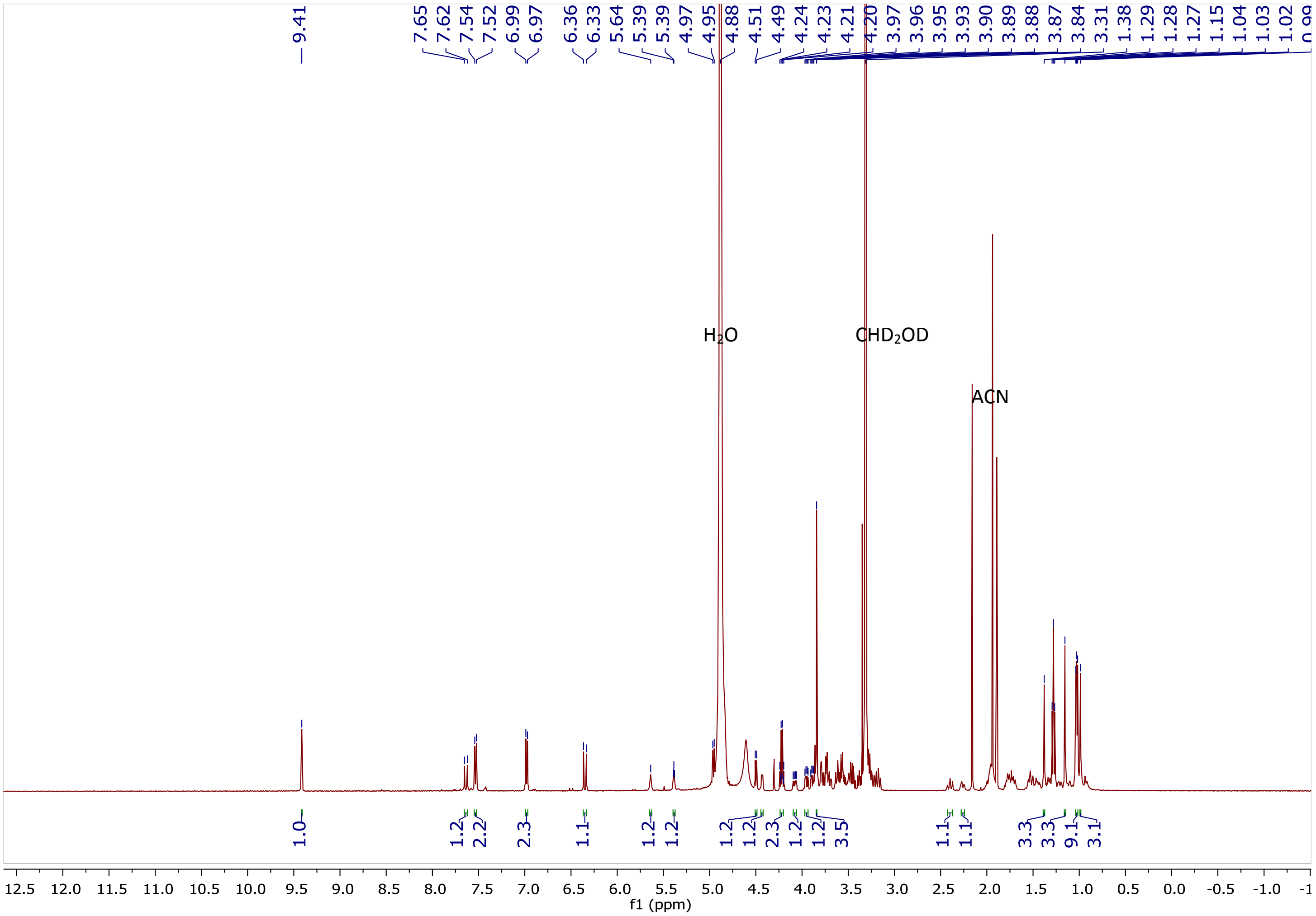

3.2 ^{13}C NMR spectrum of **3** (measured in CD_3OD)

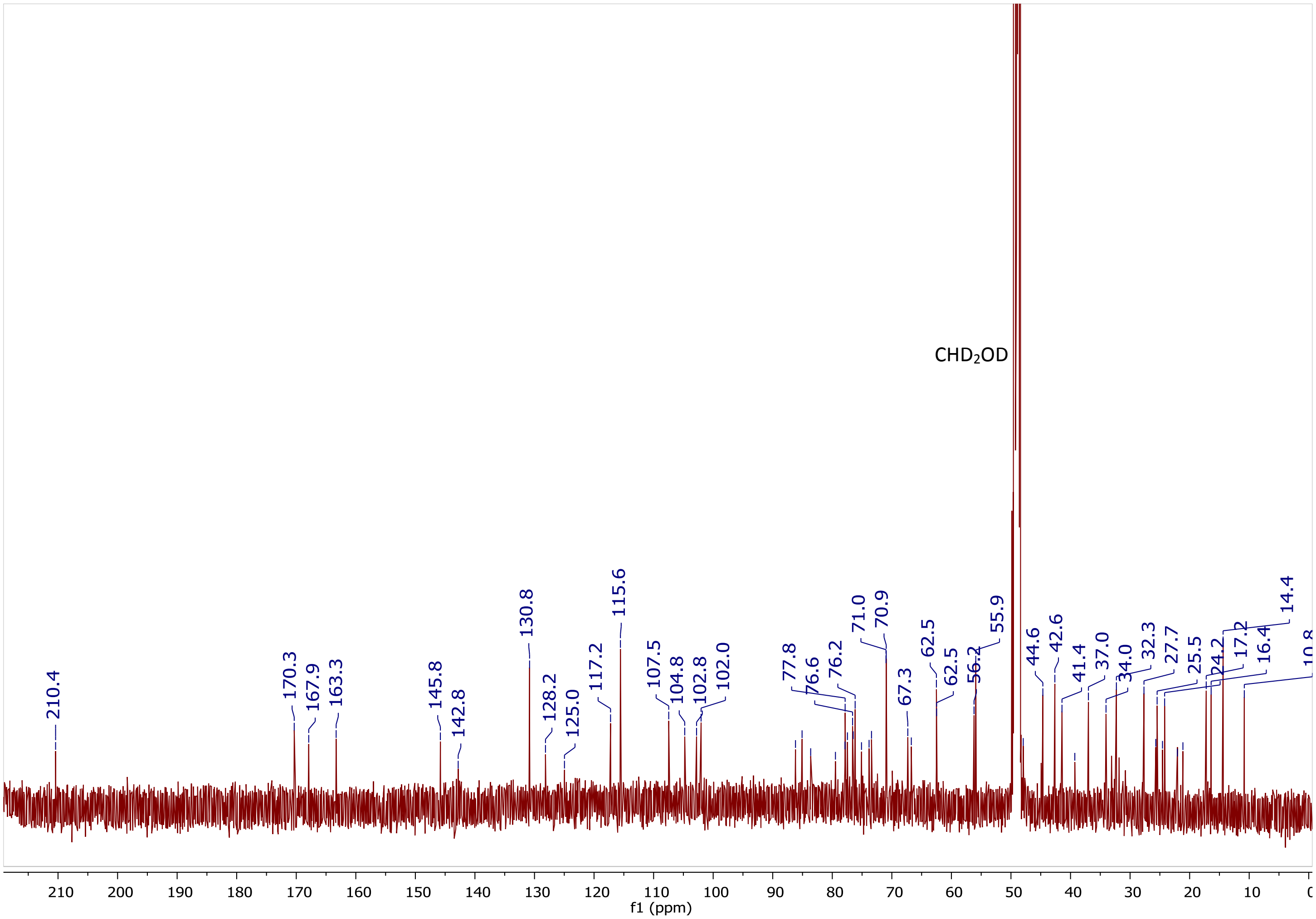

4. ^{1}H NMR spectra of 4 (measured in $CDCl_3$)

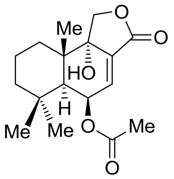

4

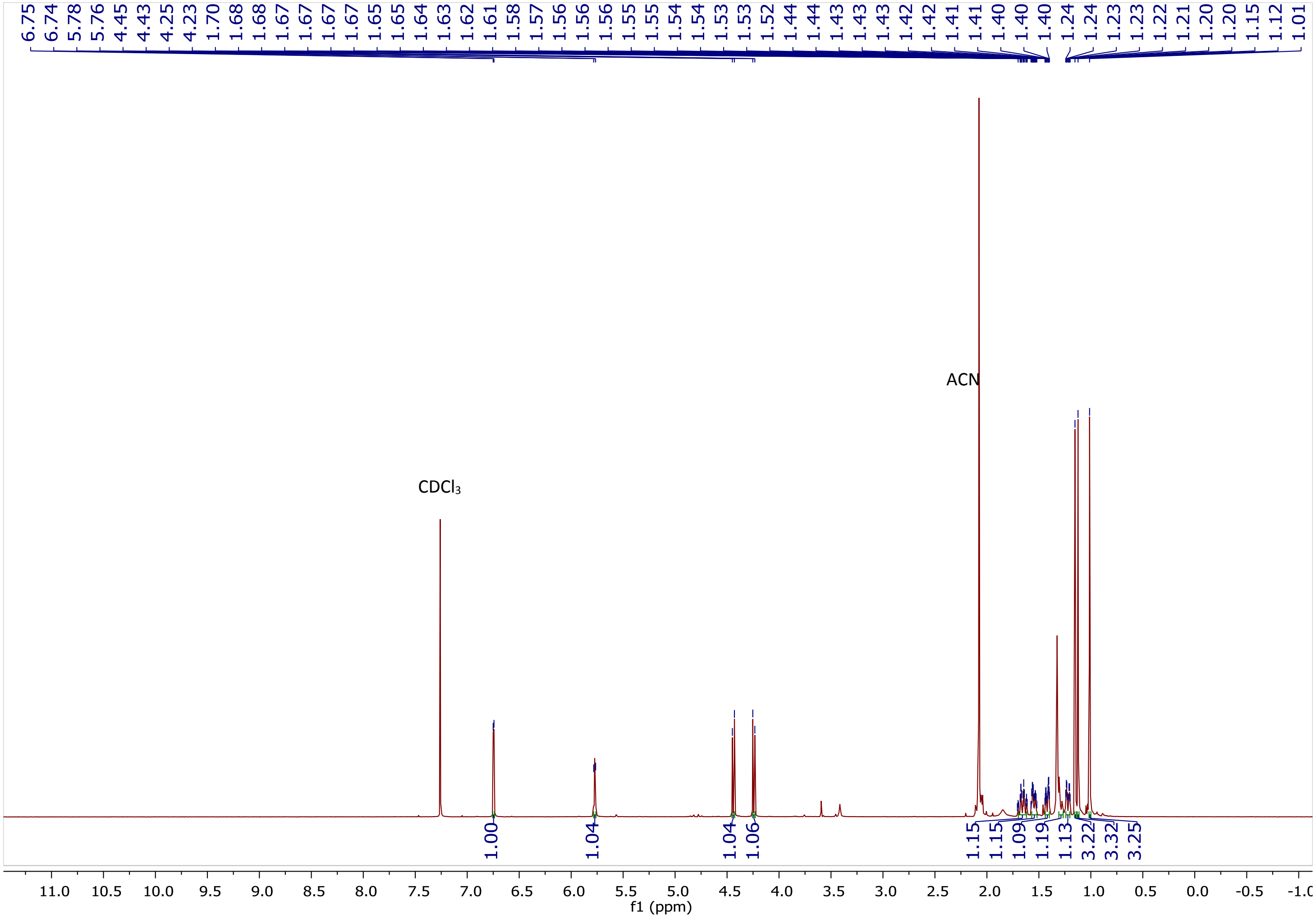

5. Purification of compounds 1-4 on C_{18} HPLC

5.1 Compound **1**

Analytical C_{18} Cogent Bidentate column (4 *μm*, 76 X 4.6 mm) eluted with a solvent gradient from $CH_3CN:H_2O$, 40:60 to 70:30 from 0 to 25 min, to 90:10 from 25 to 35 min, to 100:0 from 35 to 40 min, ending with 100% CH_3CN from 40 to 45 min at a flow rate of 1.5 mL/min.

t_{R1} 6.5 min

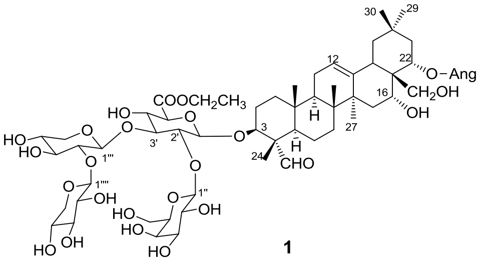

1

UV

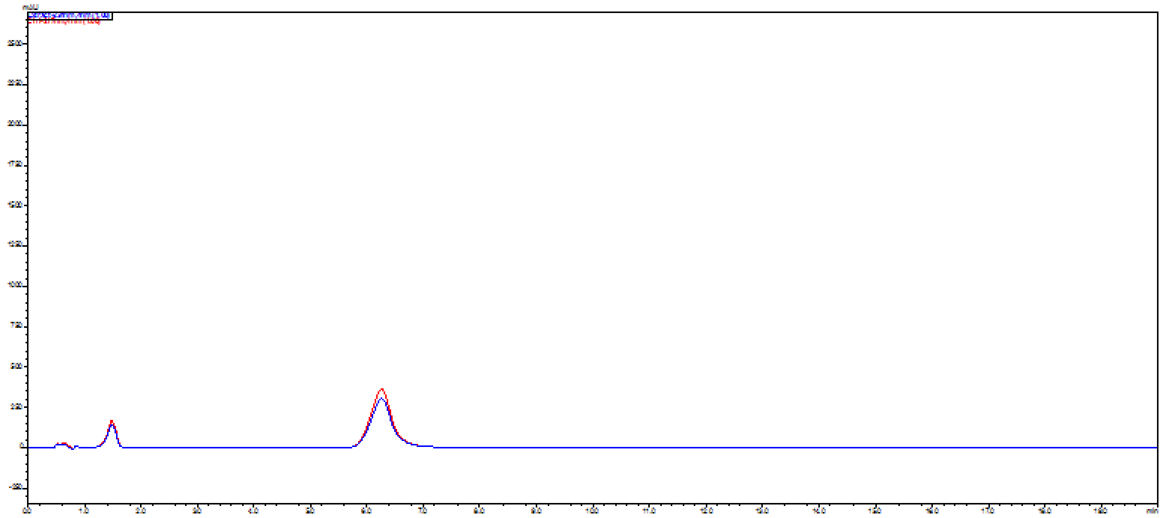

ELSD

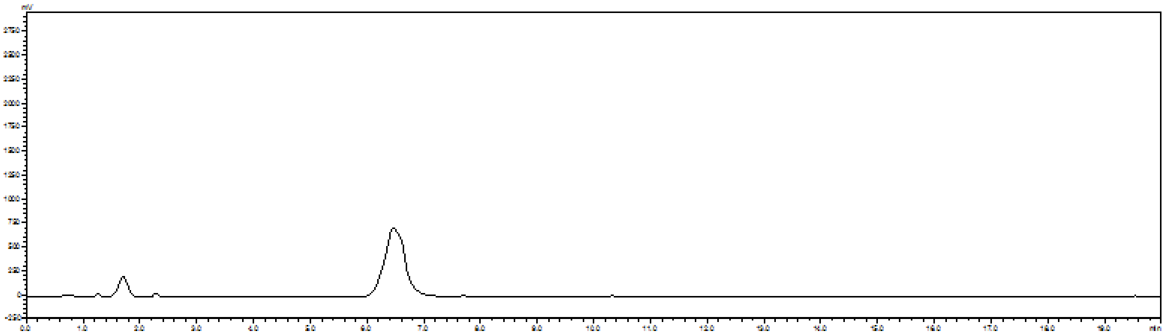

5.2 Compound **2**

Analytical C_{18} Cogent Bidentate column (4 *μm*, 76 X 4.6 mm) eluted with a solvent gradient from CH_3CN:H_2O, 40:60 to 70:30 from 0 to 25 min, to 90:10 from 25 to 35 min, to 100:0 from 35 to 40 min, ending with 100% CH_3CN from 40 to 45 min at a flow rate of 1.5 mL/min.

t_{R2} 9.5 min

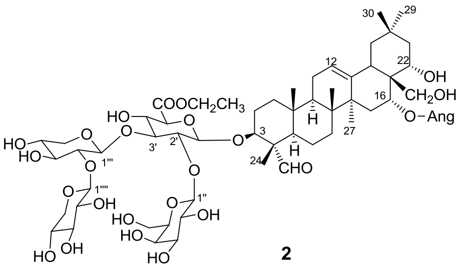

2

UV

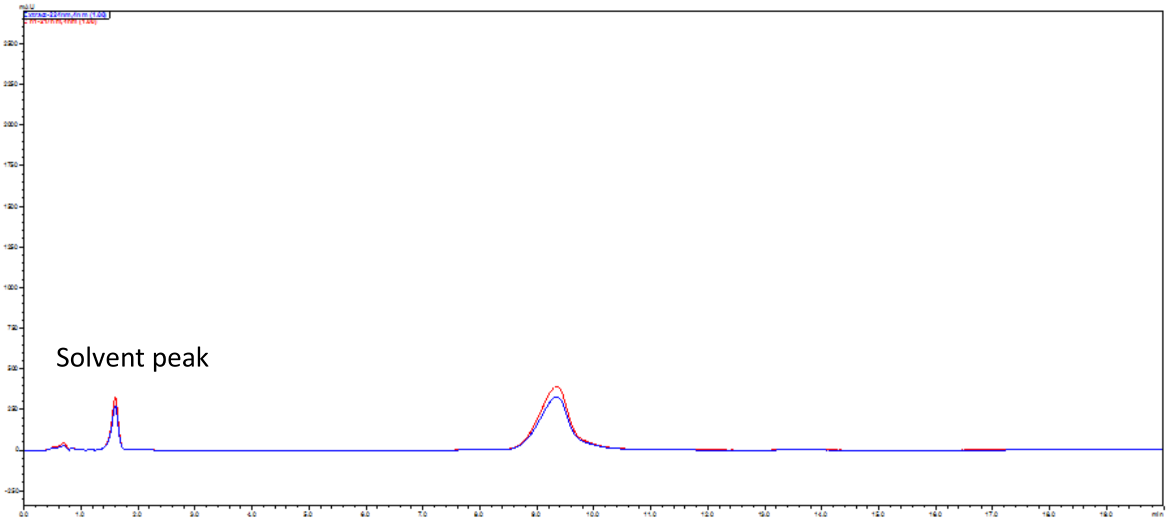

ELSD

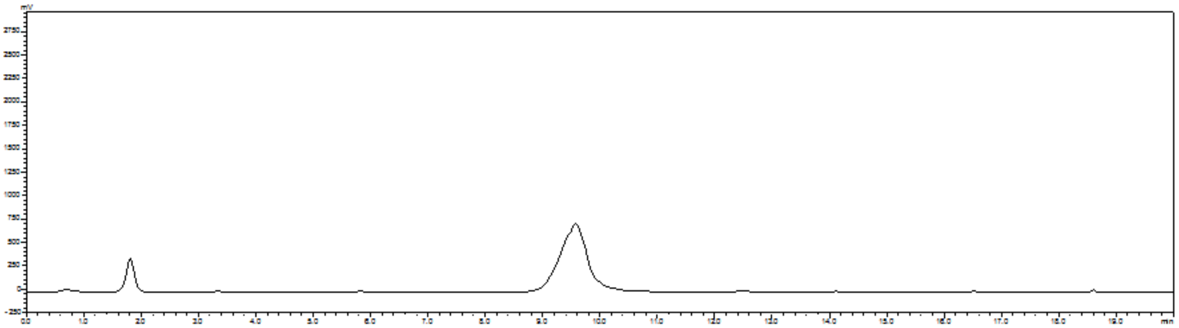

5.3 Compound **3**

Analytical C_{18} Cogent Bidentate column (4 *μm*, 76 X 4.6 mm) eluted with a solvent gradient from $CH_3CN:H_2O$, 50:50 to 65:35 from 0 to 15 min, to 80:20 from 15 to 25 min, to 100:0 from 25 to 35 min, ending with 100% CH_3CN from 35 to 45 min at a flow rate of 1.5 mL/min.

t_{R3} 17.5 min

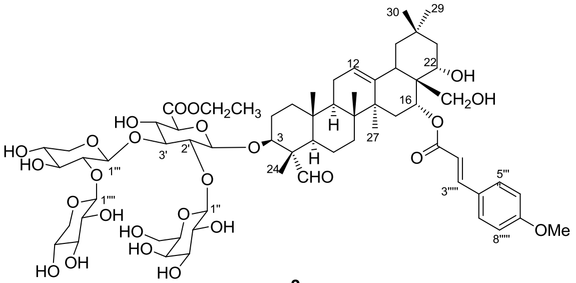

3

UV

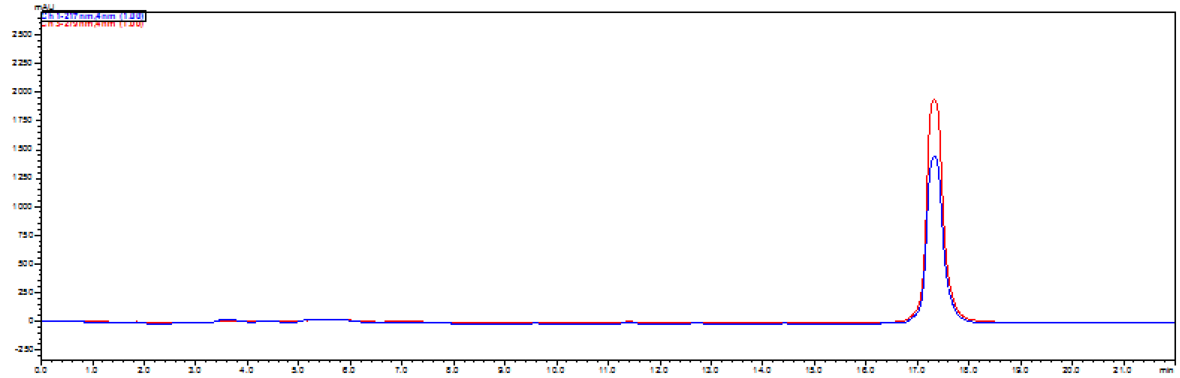

ELSD

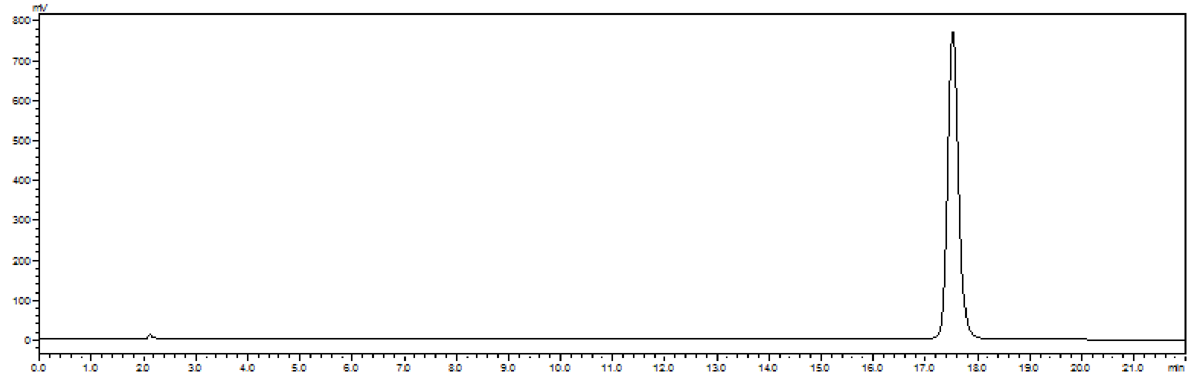

5.4 Compound **4**

Analytical C_{18} Cogent Bidentate column (4 *μm*, 76 X 4.6 mm) eluted with a solvent gradient from $CH_3CN:H_2O$, 70:30 to 75:25 from 0 to 10 min, to 100:0 from 10 to 20 min, ending with 100% CH_3CN from 20 to 30 min at a flow rate of 1.5 mL/min.

t_{R4} 11.5 min

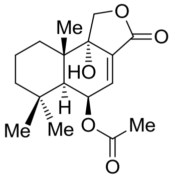

4

UV

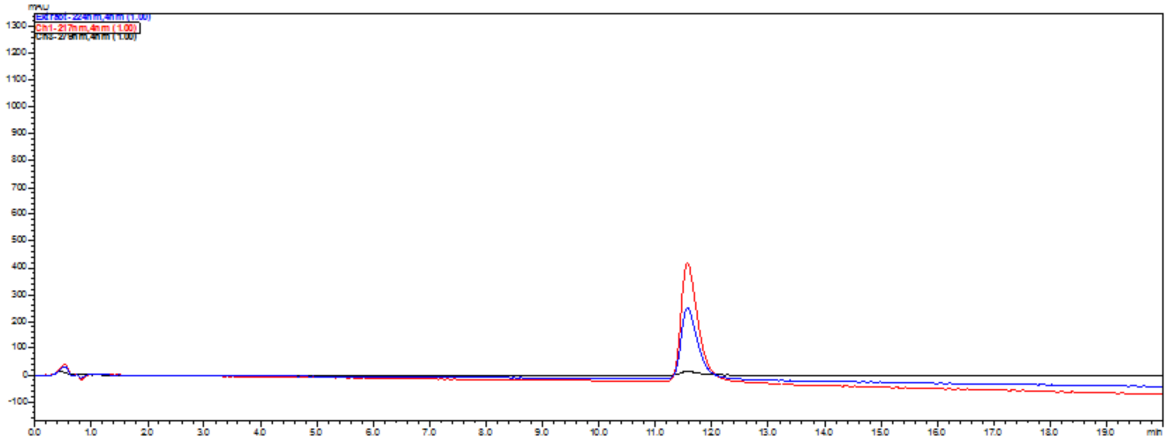

ELSD

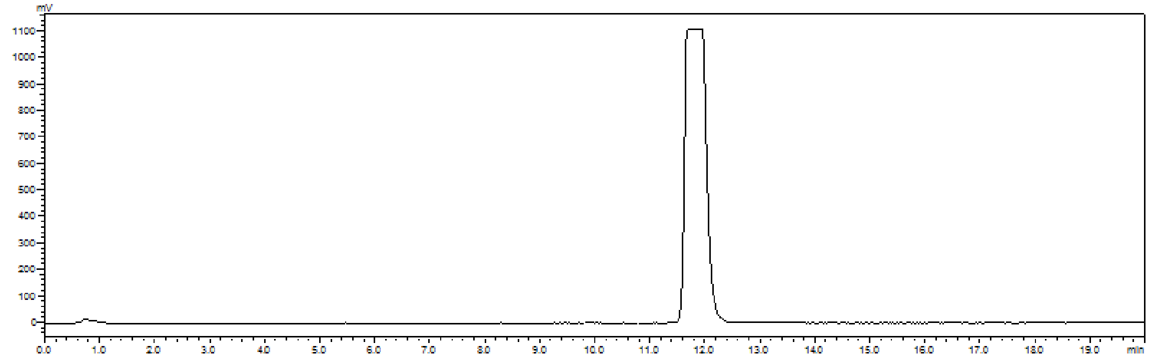

6. HPLC analysis of sugar derivatives

D-Gal derivative (t_R =25.35min)

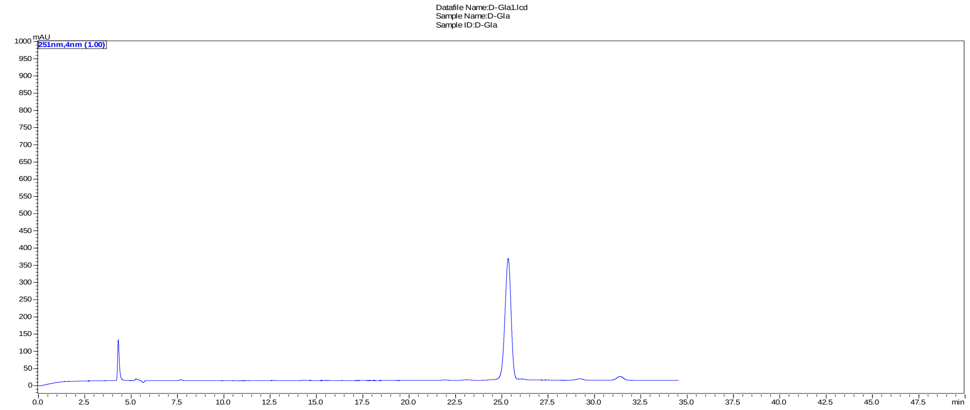

L-Gal derivative (t_R = 26.08 min)

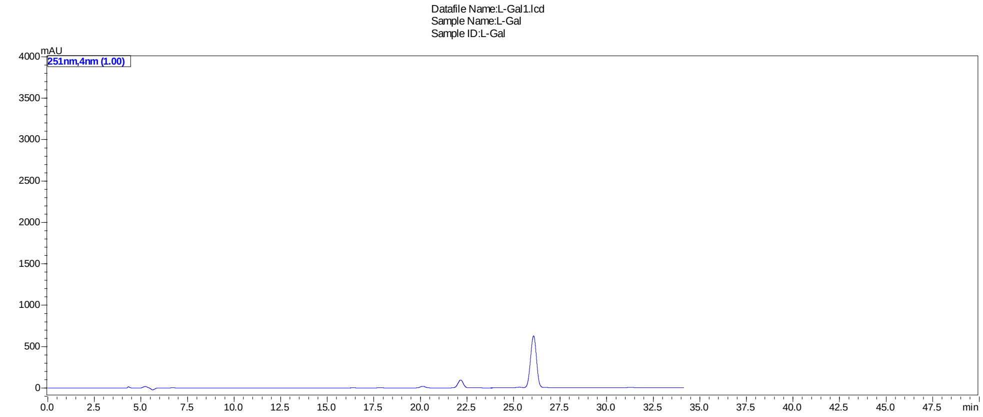

L-Glu derivative (t_R =26.76 min)

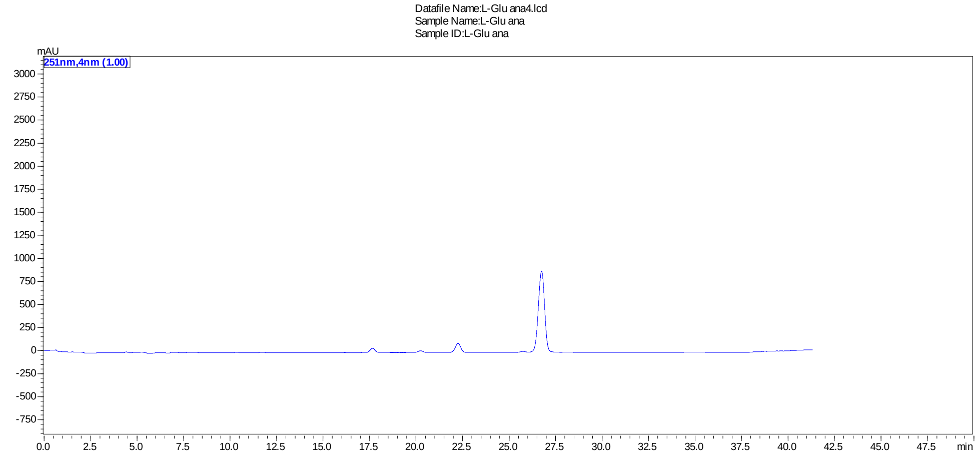

D-Glu derivative (t_R = 28.04 min)

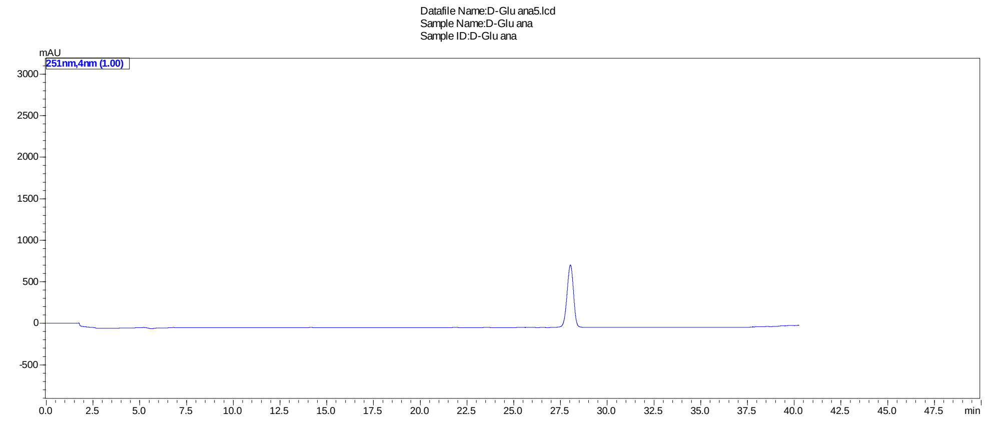

L-GlcA derivative (D-GlcA + D-cysteine methyl ester) (t_R = 28.26 min)

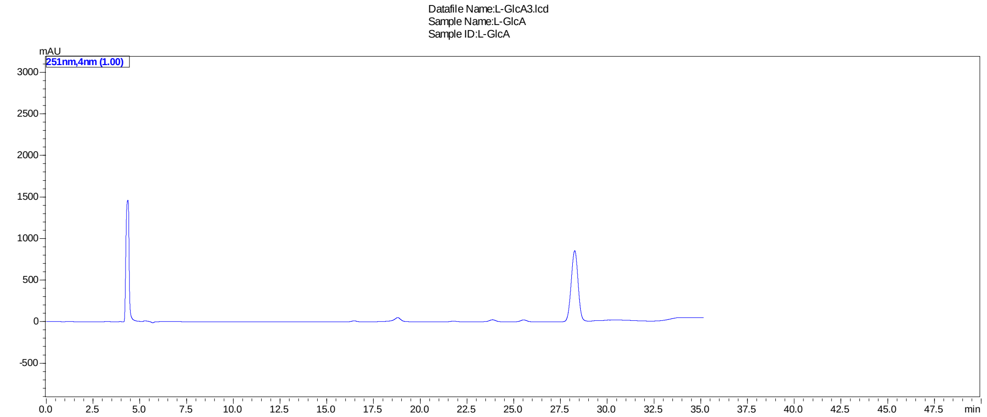

D-GlcA derivative (t_R =29.03 min)

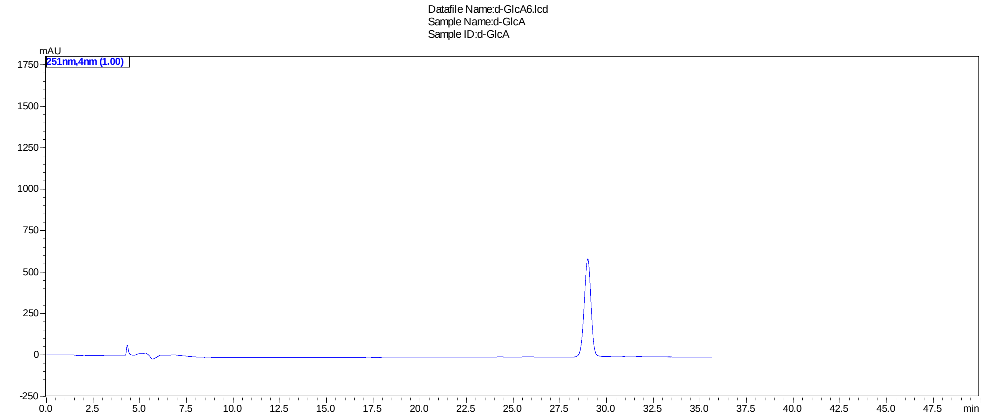

L-Xyl derivative (t_R =20.02 min)

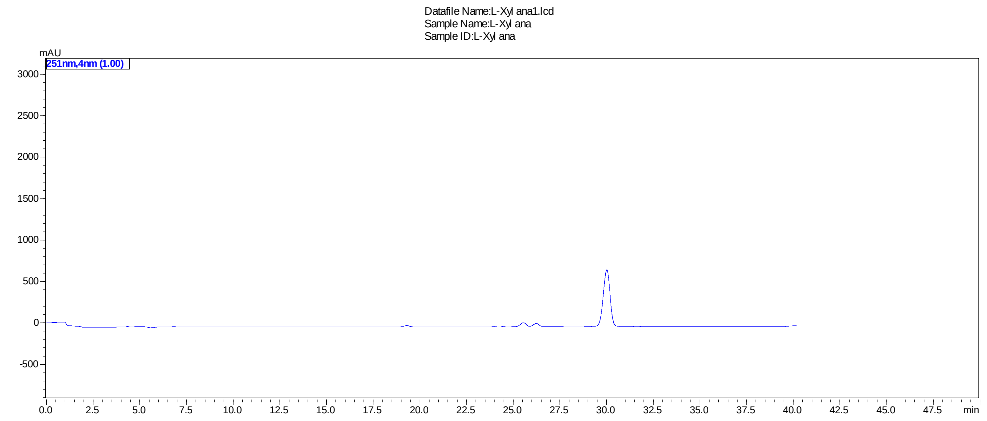

L-Ara derivative (t_R =30.28 min)

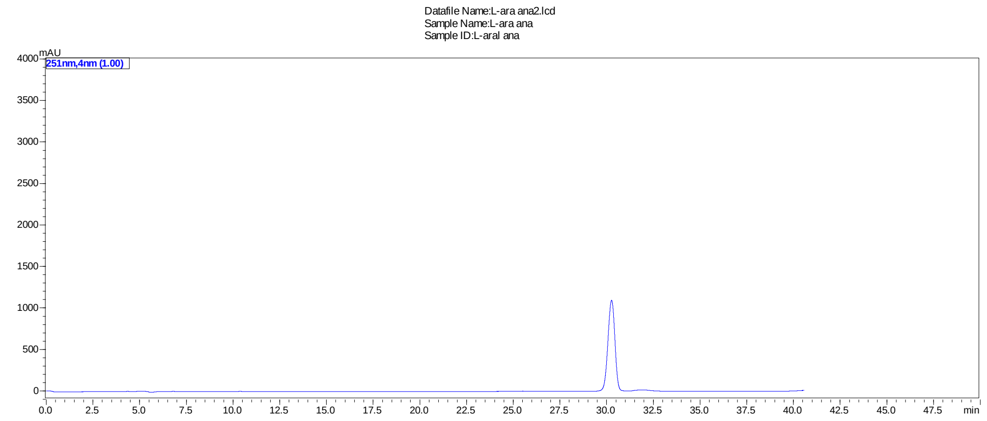

D-Xyl derivative (t_R = 31.32 min)

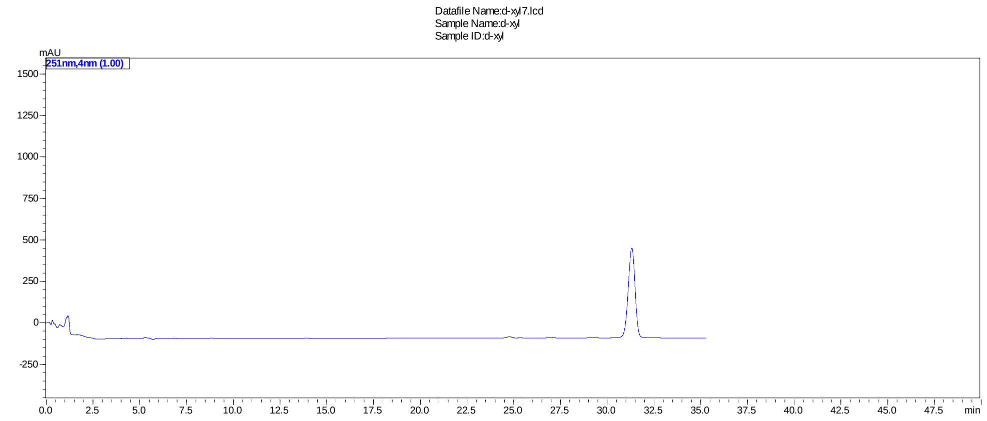

D-Ara derivative (t_R =31.71 min)

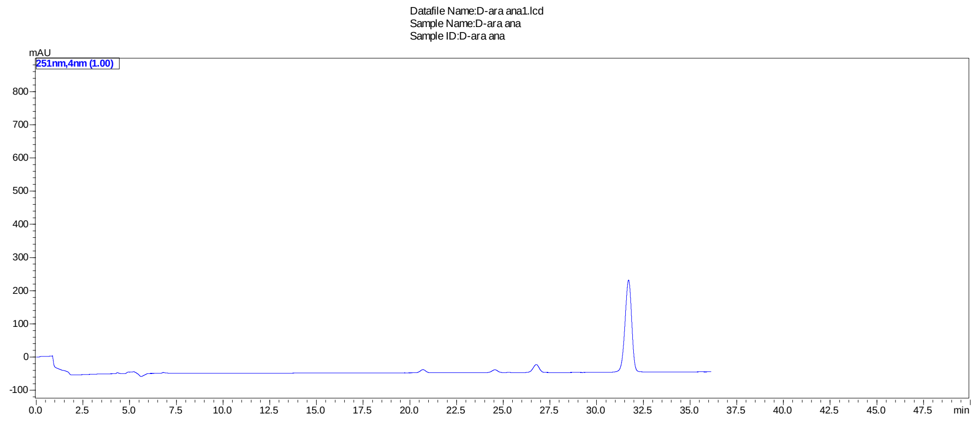

Hydrolysis products' derivatives: peaks at 25.29, 29.12, 31.25 min coincided with D-Gal, D-GlcA, D-Xyl derivatives

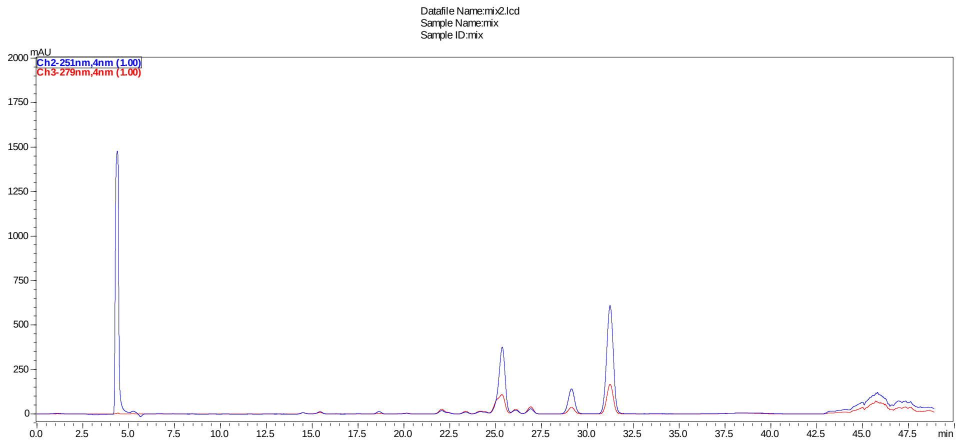

Hydrolysis products' derivatives + D-Gal derivative

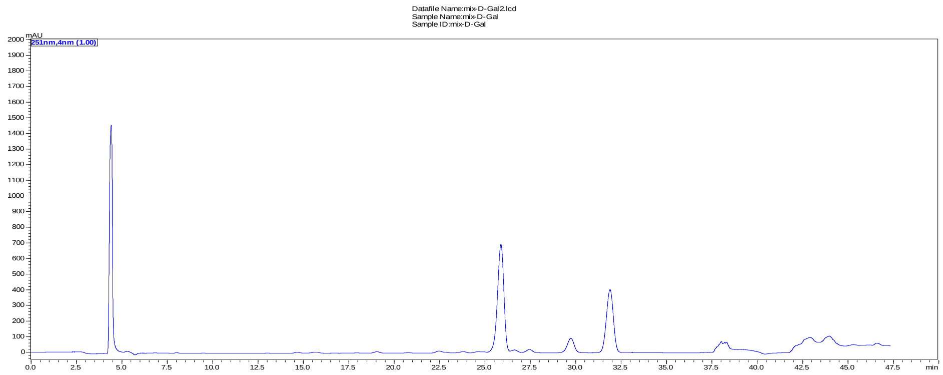

Hydrolysis products' derivatives + D-GlcA derivatives

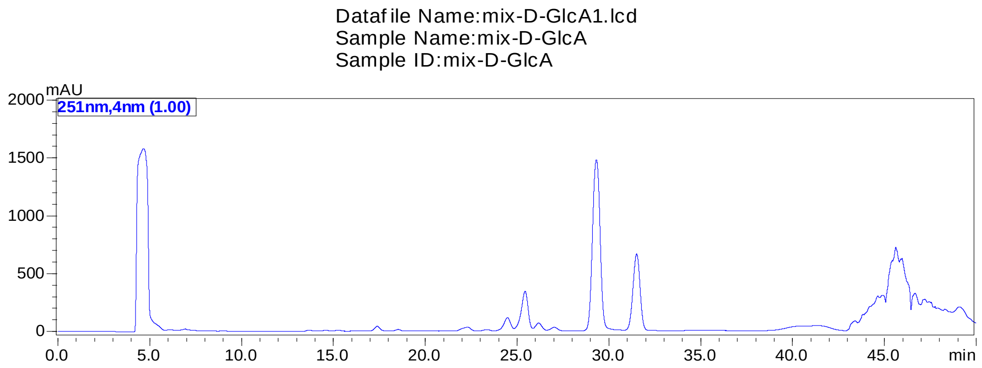

Hydrolysis products' derivatives + D-GlcA & D-Xyl derivatives

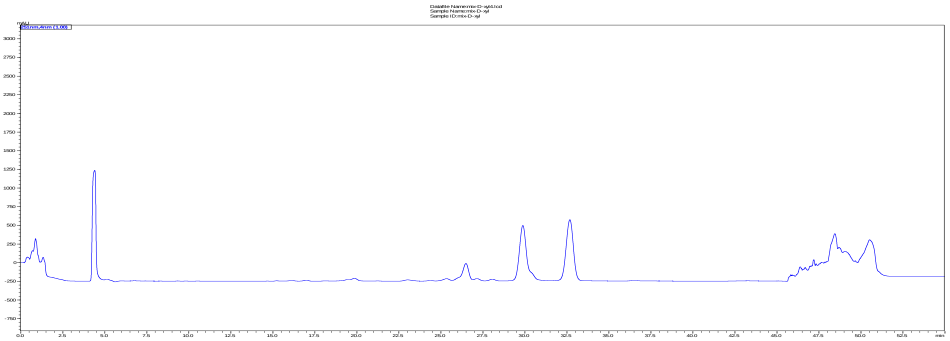

7. NMR spectra of some standard D-sugar derivatives (measured in CD_3OD)

7.1 NMR spectra of standard D-galactose derivative

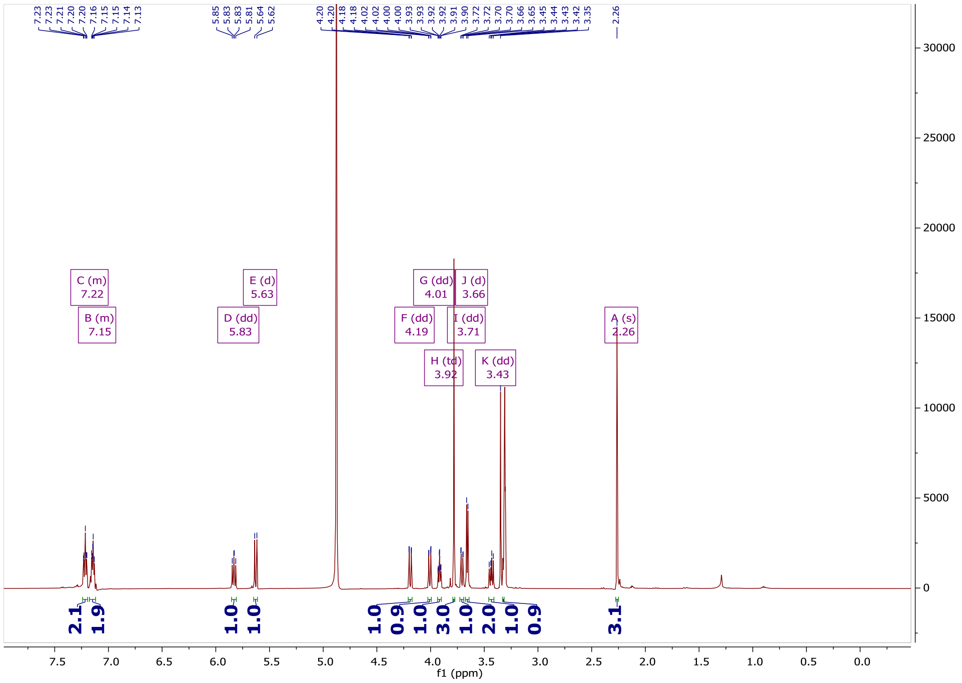

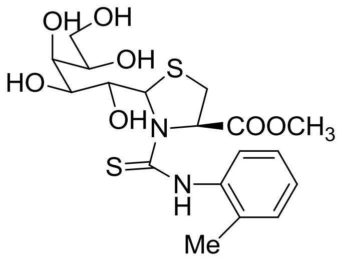

D-Gal derivative

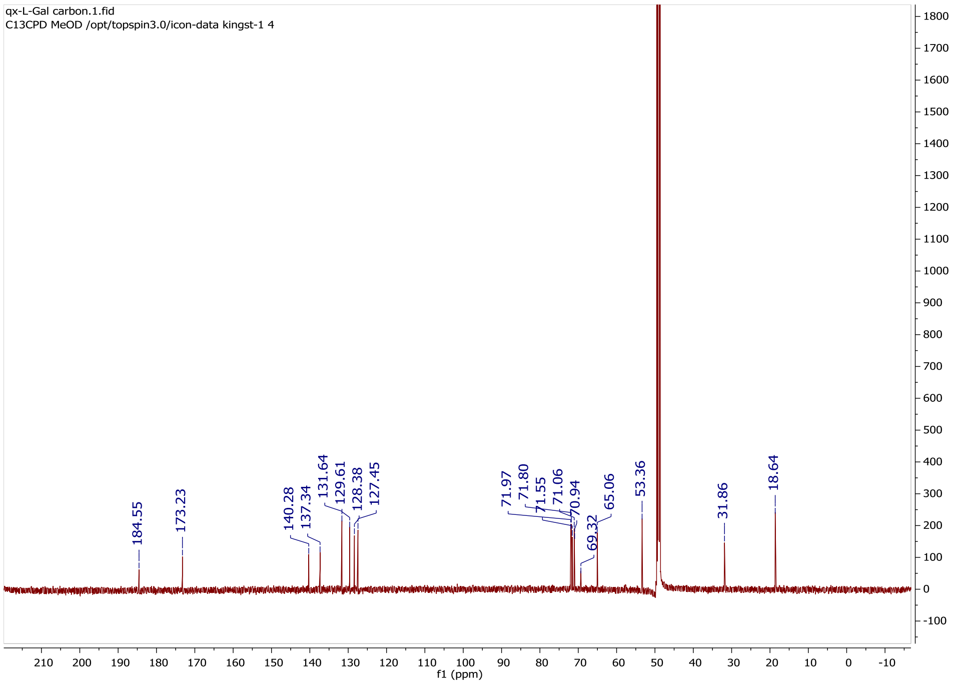

7.2 NMR spectra of standard D -xylose derivative

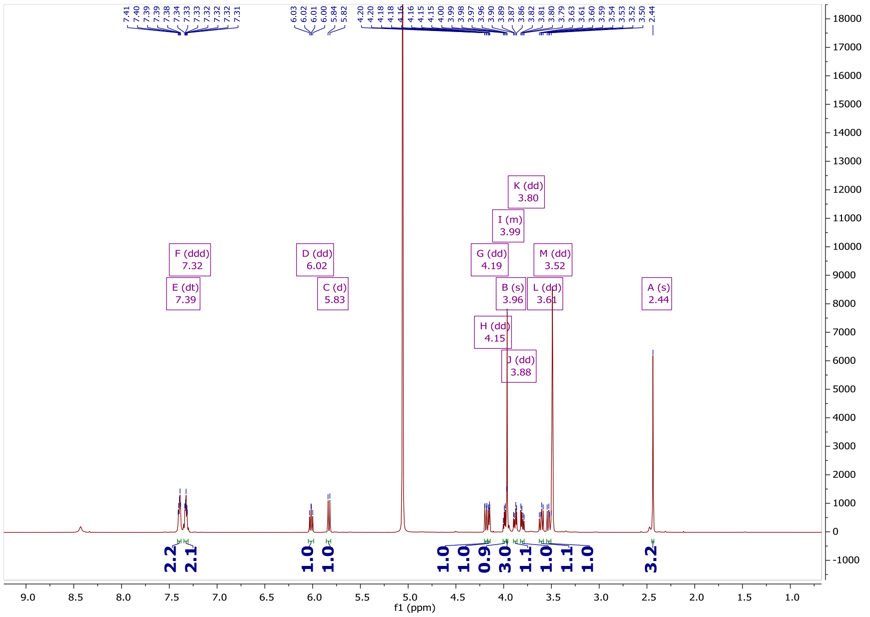

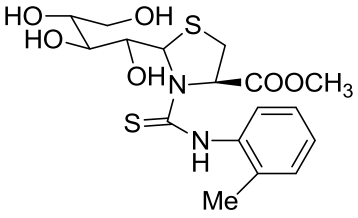

D-Xyl derivative

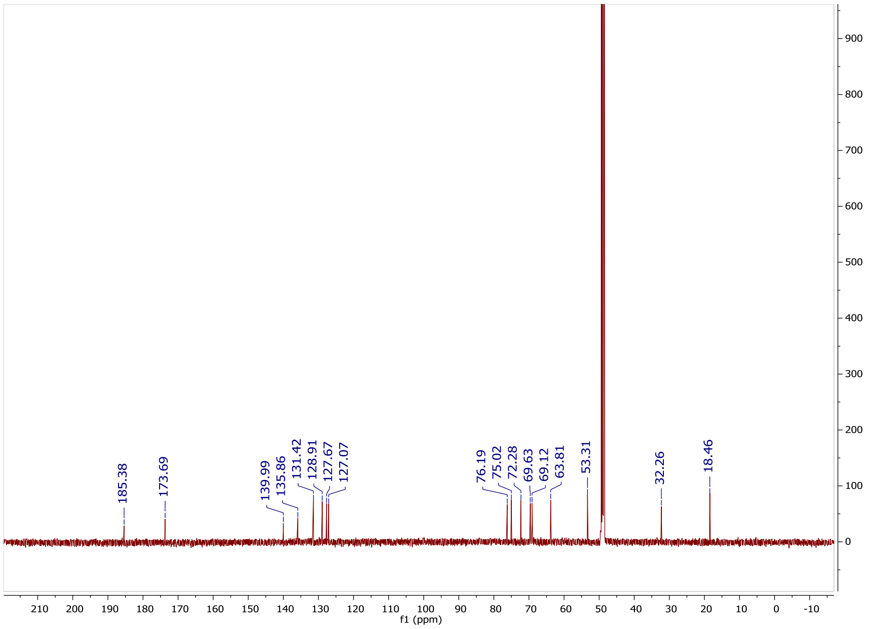

7.3 NMR spectra of standard D-Glucuronic acid derivative

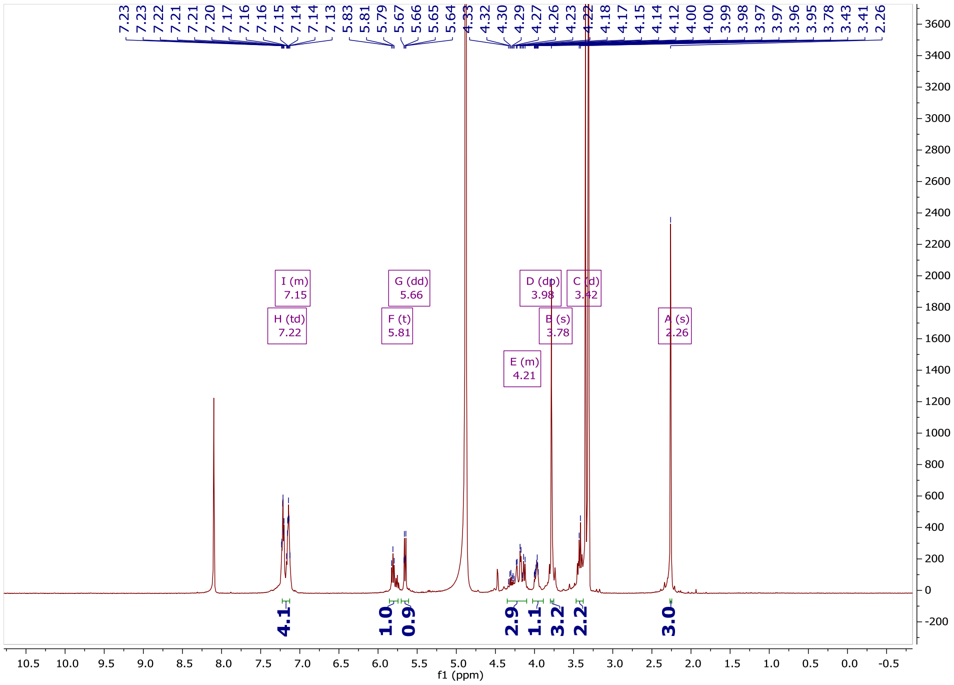

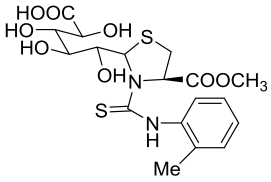

D-Glu derivative

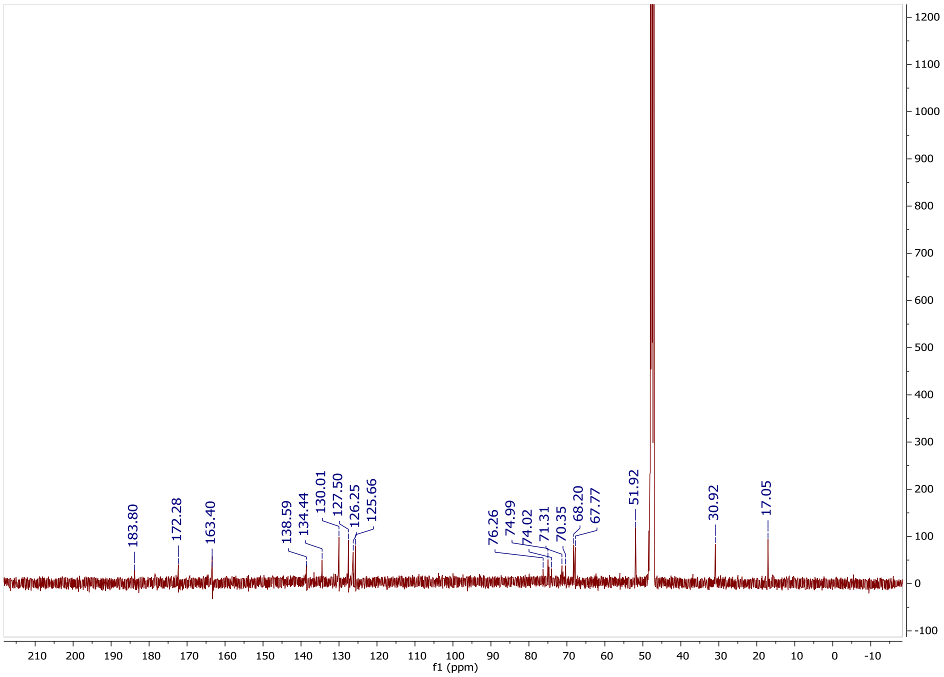

Supplement: Supplementary file 1 — Supplementary material 1 (PDF 2013 kb) [file 13659_2015_83_MOESM1_ESM.pdf]
